# Multivalent S2-based vaccines provide broad protection against SARS-CoV-2 variants of concern and pangolin coronaviruses

**DOI:** 10.1016/j.ebiom.2022.104341

**Published:** 2022-11-11

**Authors:** Peter J. Halfmann, Steven J. Frey, Kathryn Loeffler, Makoto Kuroda, Tadashi Maemura, Tammy Armbrust, Jie E. Yang, Yixuan J. Hou, Ralph Baric, Elizabeth R. Wright, Yoshihiro Kawaoka, Ravi S. Kane

**Affiliations:** a Influenza Research Institute, Department of Pathobiological Sciences, School of Veterinary Medicine, University of Wisconsin, Madison, WI, 53711, USA; b School of Chemical & Biomolecular Engineering, Georgia Institute of Technology, Atlanta, GA, 30332, USA; c Department of Biochemistry, University of Wisconsin, Madison, WI, 53706, USA; d Cryo-EM Research Center, Department of Biochemistry, University of Wisconsin, Madison, WI, 53706, USA; e Midwest Center for Cryo-Electron Tomography, Department of Biochemistry, University of Wisconsin, Madison, WI, 53706, USA; f Department of Epidemiology, University of North Carolina at Chapel Hill, Chapel Hill, NC, 27599, USA; g Department of Microbiology and Immunology, University of North Carolina at Chapel Hill, Chapel Hill, NC, 27599, USA; h Division of Virology, Department of Microbiology and Immunology, Institute of Medical Science, University of Tokyo, Tokyo 108-8639, Japan; i Wallace H. Coulter Department of Biomedical Engineering, Georgia Institute of Technology, Atlanta, GA, 30332, USA

**Keywords:** SARS-CoV-2, Coronavirus, COVID-19, Vaccine

## Abstract

**Background:**

The COVID-19 pandemic continues to cause morbidity and mortality worldwide. Most approved COVID-19 vaccines generate a neutralizing antibody response that primarily targets the highly variable receptor-binding domain (RBD) of the SARS-CoV-2 spike (S) protein. SARS-CoV-2 “variants of concern” have acquired mutations in this domain allowing them to evade vaccine-induced humoral immunity. Recent approaches to improve the breadth of protection beyond SARS-CoV-2 have required the use of mixtures of RBD antigens from different sarbecoviruses. It may therefore be beneficial to develop a vaccine in which the protective immune response targets a more conserved region of the S protein.

**Methods:**

Here we have developed a vaccine based on the conserved S2 subunit of the S protein and optimized the adjuvant and immunization regimen in Syrian hamsters and BALB/c mice. We have characterized the efficacy of the vaccine against SARS-CoV-2 variants and other coronaviruses.

**Findings:**

Immunization with S2-based constructs elicited a broadly cross-reactive IgG antibody response that recognized the spike proteins of not only SARS-CoV-2 variants, but also SARS-CoV-1, and the four endemic human coronaviruses. Importantly, immunization reduced virus titers in respiratory tissues in vaccinated animals challenged with SARS-CoV-2 variants B.1.351 (beta), B.1.617.2 (delta), and BA.1 (omicron) as well as a pangolin coronavirus.

**Interpretation:**

These results suggest that S2-based constructs can elicit a broadly cross-reactive antibody response resulting in limited virus replication, thus providing a framework for designing vaccines that elicit broad protection against coronaviruses.

**Funding:**

10.13039/100000002NIH, 10.13039/100009619Japan Agency for Medical Research and Development, Garry Betty/ V Foundation Chair Fund, and 10.13039/100000001NSF.

**Research in context**

**Evidence before this study**

Licensed SARS-CoV-2 vaccines target the spike (S) protein and primarily generate a neutralizing antibody response targeting the S1 domain, particularly the receptor-binding domain (RBD). These vaccines have been highly effective at preventing severe illness from SARS-CoV-2, but their efficacy has declined against new SARS-CoV-2 variants relative to the early isolates. Recent attempts to extend the breadth of protection beyond SARS-CoV-2 have used mixtures of RBD antigens from different sarbecoviruses. The S2 domain is a potential alternative antigen for broadly protective vaccines, since it is known to be more conserved than the S1 domain and is the target of neutralizing and cross-reactive antibodies.

**Added value of this study**

In this study, we designed a SARS-CoV-2 S2-based nanoparticle vaccine that shows efficacy *in vivo*. This vaccine construct elicited broadly cross-reactive antibodies that bound S proteins from SARS-CoV-2 variants as well as more distant coronaviruses. After immunization, the S2-based vaccine significantly reduced virus titers in hamsters challenged with SARS-CoV-2 variants B.1.351 (beta), B.1.617.2 (delta), and BA.1 (omicron), as well as from a pangolin coronavirus.

**Implications of all the available evidence**

Our results suggest that the S2 subunit should be considered in the development of next-generation coronavirus vaccines designed to provide broader protection against future SARS-CoV-2 variants and other zoonotic coronaviruses with pandemic potential.

## Introduction

**Fig. 1:**
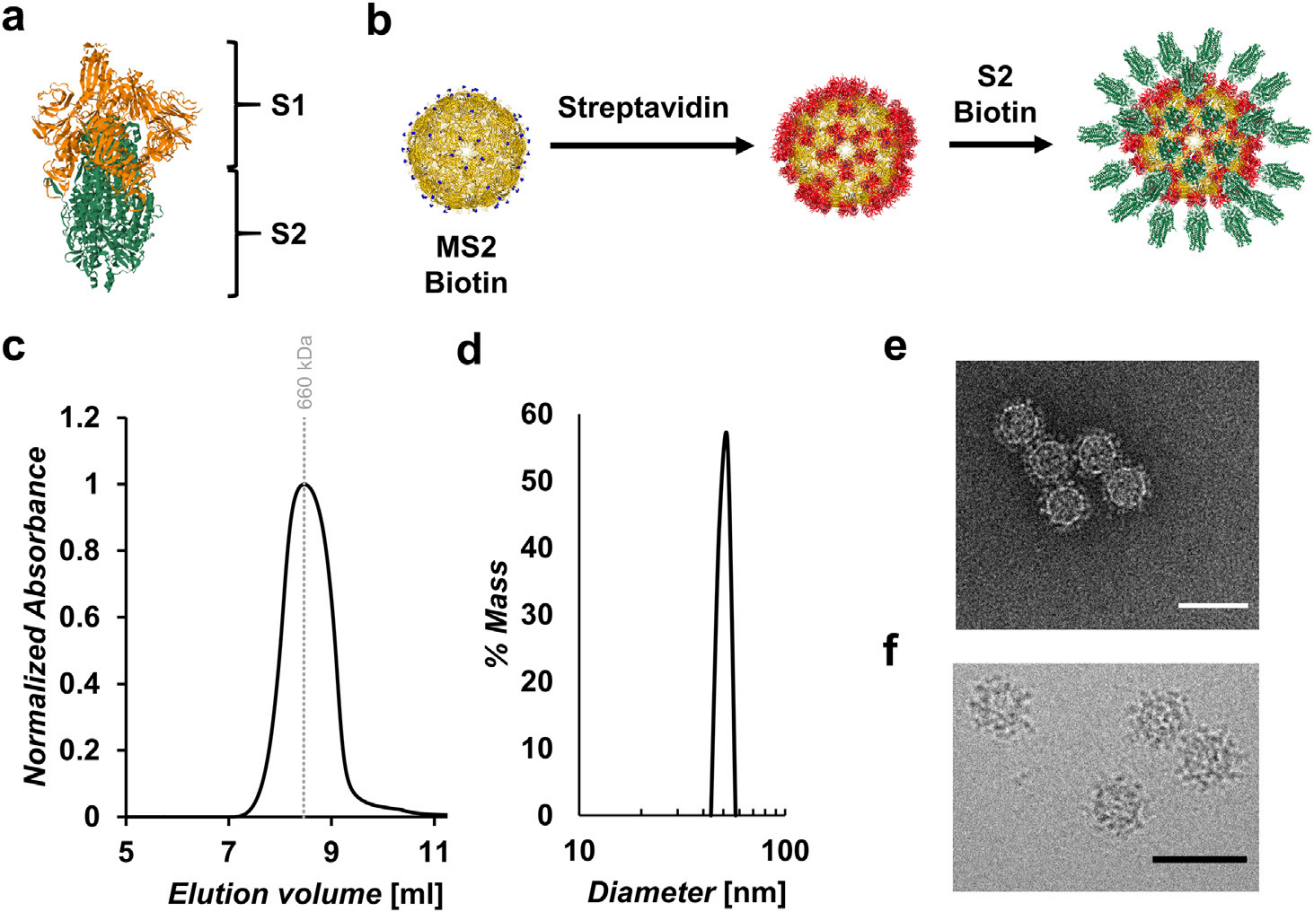
**Assembly of VLP-S2 and characterization of MS2-SA VLP.(a)** The SARS-CoV-2 spike ectodomain (PDB: 6XKL). The S1 subunit is highlighted in orange and the S2 subunit is highlighted in green. **(b)** Scheme illustrating the assembly of VLP-S2, where biotinylated MS2 (yellow, PDB: 2MS2) is added to streptavidin to create the VLP. S2 biotinylated at the C-terminus (green; PDB: 6XKL) is mixed with the VLP to create the VLP-S2. **(c)** Size exclusion chromatography trace for MS2-SA VLP. The vertical gray line represents the peak elution volume of the molecular weight standard thyroglobulin (660 kDa). The column void volume is 7.2 mL. **(d)** Characterization of the MS2-SA VLP by dynamic light scattering. **(e)** Negative-stain transmission electron micrograph of MS2-SA VLPs. Scale bar = 50 nm. **(f)** Cryo-EM of vitrified MS2-SA VLP. Scale bar = 50 nm.

The novel coronavirus SARS-CoV-2 has had an astounding impact on world health since it was first identified in December 2019. In fact, over 6.3 million people worldwide have died as a result of contracting the virus, and many more have been infected.^1^ A wide variety of SARS-CoV-2 vaccine candidates have been developed, including nucleic acid-based vaccines, viral vector-based vaccines, subunit vaccines, and inactivated vaccines.^2^ In particular, a number of vaccines that target the SARS-CoV-2 spike (S) protein have been authorized for use.^3^, ^4^, ^5^, ^6^, ^7^, ^8^ The spike protein is a glycoprotein displayed on the surface of the SARS-CoV-2 virus that allows the virus to bind to host cells through its S1 subunit and fuse to the host cell membrane through its S2 subunit (Fig. 1a). This key role in viral attachment and entry into cells has made the S protein an effective vaccine target and several S protein-based vaccines have been shown to successfully prevent SARS-CoV-2 infection.^3^, ^4^, ^5^, ^6^ However, the threat of emerging variants that escape vaccine-mediated immunity is a cause for concern,^9^, ^10^, ^11^, ^12^ as exemplified by the recent waves of infections caused by the B.1.617.2 (delta) and BA.1 (omicron) variants, with newer variants like BA.2, BA.4, and BA.5 continuing to emerge and displace ancestral strains.^13^, ^14^, ^15^ In addition, some zoonotic coronaviruses have been identified to have pandemic potential^16^^,^^17^ and others such as SARS-CoV-1 and MERS-CoV are already known to cause severe disease in humans. Therefore, a broadly protective coronavirus vaccine may prove useful.

The S2 subunit of the spike protein has been identified as a promising target for a broadly protective coronavirus vaccine, as it is considerably more conserved than the S1 subunit. In particular, the functionally important fusion peptide region in the S2 subunit may be an attractive target for cross-reactive antibodies.^18^, ^19^, ^20^ Antibodies targeting the S2 subunit have been found to neutralize SARS-CoV-2.^21^, ^22^, ^23^, ^24^, ^25^, ^26^, ^27^, ^28^ Even S2-specific antibodies that do not directly neutralize SARS-CoV-2 may mitigate pathological burden through Fc effector functions.^29^ Furthermore, antibodies targeting the S2 subunit have been found to be cross-reactive among coronaviruses.^30^, ^31^, ^32^ For instance, Wang et al. isolated two human monoclonal antibodies from immunized humanized mice that displayed cross-reactivity against the spike proteins of betacoronaviruses including SARS-CoV-1, SARS-CoV-2, MERS-CoV, and HCoV-OC43.^32^ Some cross-reactive S2-specific antibodies are also capable of neutralizing across coronavirus types.^22^, ^23^, ^24^, ^25^^,^^33^ For example, Pinto et al.^24^ described a human S2-specific monoclonal antibody that showed neutralization activity against not only authentic SARS-CoV-2 but also against viruses pseudotyped with SARS-CoV-1 S, Pangolin Guangdong 2019 S, MERS-CoV S, and OC43 S. Collectively, these findings indicate that S2-based vaccines may provide broad protection against coronaviruses.

Recently, a SARS-CoV-2 S2 immunogen was evaluated by Ravichandran et al.^34^ They found that compared to the spike ectodomain and other S1-based antigens, the S2 immunogen generated relatively low anti-spike antibody titers and weak SARS-CoV-2 neutralization titers. We hypothesized that the multivalent display of the S2 subunit with an appended C-terminal trimerization domain to promote its stability – i.e., the generation of nanoparticle scaffolds presenting multiple copies of the stabilized S2 subunit trimers – might help elicit a strong response against S2, as the multivalent display of antigens has been shown to generate strong immune responses.^35^ Moreover, we hypothesized that such an immunogen without the immunodominant S1 subunit would elicit a strong response targeting the S2 subunit that would have otherwise been directed towards the S1 subunit. To that end, we generated virus-like particles (VLPs) that multivalently displayed either the S2 subunit of the SARS-CoV-2 spike protein or an S2 variant designed to prevent potential proteolytic cleavage at the S2’ cut site. After characterizing the VLP-S2 constructs *in vitro*, we used them to vaccinate hamsters. The immunized hamsters showed significantly lower viral titers in the lungs and nasal turbinates after challenge with an early isolate of SARS-CoV-2 compared to control hamsters. Sera from the hamsters immunized with VLP-S2 showed substantial cross-reactive IgG antibody recognition of the spike proteins of SARS-CoV-2 variants, SARS-CoV-1, and the four endemic human coronaviruses. Most importantly, immunization also significantly reduced virus titers in the respiratory tissues of hamsters challenged with SARS-CoV-2 variants B.1.351 (beta), B.1.617.2 (delta), and BA.1 (omicron) as well as with a pangolin coronavirus. Immunization inhibited virus replication in the lungs of VLP-S2-vaccinated mice challenged with a mouse-adapted SARS-CoV-2 and elicited a broad neutralizing response. These results suggest that S2-based immunogens are an attractive approach to design broadly protective coronavirus vaccines.

## Methods

### Expression and purification of SARS-CoV-2 S2 and S2_mutS2’_ proteins

The gene encoding the S2 subunit of the SARS-COV-2 HexaPro^36^ spike protein (residues 686 to 1208) with an N-terminal mouse Ig Kappa signal peptide and C-terminal T4 fibritin trimerization domain, AviTag, and his-tag was cloned into pcDNA3.1(-) between the NcoI and XhoI restriction sites by Gene Universal, Inc. (Newark, DE). The S2_mutS2’_ variant was created such that S2 residues 814 and 815 were mutated to glycine residues to eliminate the S2’ protease cut site. These plasmids were transfected into Expi293F cells (RRID: CVCL_D615) using the ExpiFectamine Transfection Kit (Thermo Fisher Scientific) and associated protocol. The cells were incubated for 5 days, after which the cultures were centrifuged at 5,500*×g* for 20 min. The supernatant was dialyzed into PBS and then was allowed to flow through 1 mL of HisPur Ni-NTA resin (Thermo Scientific) in a gravity flow column (G-Biosciences) that had been washed with DI water and pre-equilibrated with phosphate-buffered saline (PBS). The column was then washed with 90 column volumes of wash buffer (42 mM sodium bicarbonate, 8 mM sodium carbonate, 300 mM NaCl, 20 mM imidazole). The protein was eluted by incubating the resin in 3 mL of elution buffer (42 mM sodium bicarbonate, 8 mM sodium carbonate, 300 mM NaCl, 300 mM imidazole) for 5 min before allowing the elution buffer to flow through the column. The eluate was collected. This elution procedure was repeated twice more such that a total of 9 mL of eluate was collected. The eluate was buffer exchanged into 20 mM Tris, 20 mM NaCl, pH 8.0, to prepare for *in vitro* biotinylation. The concentrations of the protein solutions were quantified using the BCA assay (Thermo Scientific).

### Expression and purification of MS2

The following protocol regarding the expression and purification of MS2 has been previously described.^37^ The DNA sequence corresponding to a single chain dimer of MS2 coat protein with an AviTag inserted between the fourteenth and fifteenth residues of the first coat protein monomer was cloned into pET-28b between the NdeI and XhoI restriction sites by GenScript Biotech Corporation (Piscataway, NJ). This plasmid and a plasmid coding for pAcm-BirA (Avidity LLC) were co-transformed into BL21(DE3) *Escherichia coli* (*E. coli*) (New England BioLabs). The transformation was added to 5 mL of 2xYT that had been supplemented with kanamycin and chloramphenicol. This small culture was incubated in a shaking incubator overnight at 37 °C. The following morning, the 5 mL culture was added to 1 L of 2xYT that had been supplemented with kanamycin and chloramphenicol. The 1 L culture was placed in a shaking incubator at 37 °C. Once the culture's optical density reached 0.6, expression of the MS2 and BirA was induced with IPTG (1 mM; GoldBio). The culture was also supplemented with approximately 12.5 μg of biotin, and remained shaking in the incubator overnight at 30 °C. After the overnight expression, the culture was centrifuged at 7000*×g* for 7 min to pellet the cells. The cell pellet was then homogenized into 25 mL of 20 mM Tris buffer (pH 9.0) supplemented with lysozyme (0.5 mg/mL; Alfa Aesar), a protease inhibitor tablet (Sigma–Aldrich), and benzonase (125 units; EMD Millipore). The resulting cell suspension was kept on ice for 20 min while occasionally mixing. Next, sodium deoxycholate was added to a final concentration of 0.1% (w/v). The cells were kept on ice and sonicated for 3 min at an amplitude of 35% with 3 s pulses (Sonifier S-450, Branson Ultrasonics). This sonication procedure was repeated after allowing the cells to cool on ice for at least 2 min. The resulting lysate was centrifuged at 27,000*×g* for 30 min. The supernatant was collected and was centrifuged again at 12,000*×g* for 15 min. The supernatant resulting from the second centrifugation was diluted 3-fold with 20 mM Tris, pH 8.0, and filtered using a 0.45 μm bottle-top filter. The filtrate was then run through four HiScreen Capto Core 700 columns (Cytiva) in series according to the manufacturer's operating instructions, resulting in fractions that contained MS2. The fractions were run on an SDS-PAGE gel to assess MS2 purity and recovery. Fractions containing pure MS2 were pooled, concentrated using a 10 kDa MWCO centrifugal filter (Millipore Sigma), and further purified using a Superdex 200 increase 10/300 SEC column (Cytiva). The SEC fractions containing MS2 were pooled and buffer exchanged into 20 mM Tris, 20 mM NaCl, pH 8.0, in preparation for *in vitro* biotinylation. MS2 was quantified using the BCA assay (Thermo Scientific).

### Expression, refolding, and purification of streptavidin (SA)

The following protocol regarding the expression, refolding, and purification of SA has been previously described and was adapted from methods documented by Fairhead et al. and Howarth et al.^37^, ^38^, ^39^ A plasmid encoding SA (RRID: Addgene_46367, a gift from Mark Howarth) was transformed into BL21(DE3) *E. coli*. The transformation was added to 5 mL of 2xYT supplemented with ampicillin, and this small culture was grown overnight in a shaking incubator at 37 °C. The next morning the culture was added to four, 1 L shake flasks of 2xYT supplemented with ampicillin. These larger cultures were placed in a shaking incubator at 37 °C until the cultures’ OD reached 0.6, at which point the expression of streptavidin as inclusion bodies was induced with IPTG (1 mM; GoldBio), and the temperature of the incubator was reduced to 30 °C. After overnight incubation, the cultures were centrifuged at 7,000*×g* for 15 min such that there were two cell pellets. The two resulting cell pellets were each homogenized into 50 mL of resuspension buffer (50 mM Tris, 100 mM NaCl, pH 8.0) supplemented with lysozyme (1 mg/mL; Alfa Aesar) and benzonase (500 units; EMD Millipore). The homogenized cells were incubated at 4 °C for at least 30 min. After this incubation step, the cells were further homogenized and sodium deoxycholate was added to a final concentration of 0.1% (w/v) before sonicating (Sonifier S-450, Branson Ultrasonics) for 3 min with 3 s pulses at 35% amplitude. The lysed cells were then centrifuged at 27,000*×g* for 15 min. The supernatant was discarded, and the lysis procedure was repeated. When the lysis step was repeated, the incubation time at 4 °C prior to sonication was reduced to 15 min. After the lysis procedure had been performed twice, the two resulting inclusion body pellets were each suspended in 50 mL wash buffer (50 mM Tris, 100 mM NaCl, 100 mM EDTA, 0.5% (v/v) Triton X-100, pH 8.0), homogenized, sonicated for 30 s at an amplitude of 35%, and centrifuged at 27,000*×g* for 15 min. This wash procedure was repeated twice more. The resulting inclusion body pellets were then suspended in 50 mL of a second wash buffer (50 mM Tris, 10 mM EDTA, pH 8.0), homogenized, sonicated for 30 s at an amplitude of 35%, and centrifuged at 15,000*×g* for 15 min. This second wash step was performed twice. The washed inclusion body pellets were then unfolded by being homogenized into 10 mL of a 7.12 M guanidine hydrochloride solution. This solution of unfolded streptavidin in guanidine hydrochloride was stirred at room temperature for an hour, after which it was centrifuged at 12,000*×g* for 10 min. The supernatant was then added dropwise at a rate of 30 mL/h to 1L of chilled PBS that was being stirred vigorously. This rapid dilution of the streptavidin and guanidine hydrochloride allowed for the streptavidin to fold properly. The folded streptavidin in PBS was stirred overnight at 4 °C, and was then centrifuged at 7,000*×g* for 15 min to remove insoluble protein. The supernatant was filtered using a 0.45 μm bottle-top filter, and was then stirred while ammonium sulfate was slowly added to a concentration of 1.9 M. This concentration of ammonium sulfate serves to precipitate out impurities. The solution was stirred for at least 3 h at 4 °C, after which it was centrifuged at 7,000*×g* for 15 min to pellet the precipitated impurities. The supernatant was filtered using a 0.45 μm bottle-top filter, and was then stirred while ammonium sulfate was added to a total concentration of 3.68 M. This concentration of ammonium sulfate precipitates the streptavidin. The solution was stirred for at least 3 h at 4 °C before being centrifuged at 7,000*×g* for 20 min to pellet the streptavidin. The supernatant was discarded, and the pelleted streptavidin was suspended in 20 mL of Iminobiotin Affinity Chromatography (IBAC) binding buffer (50 mM Sodium Borate, 300 mM NaCl, pH 11.0). This streptavidin solution was then allowed to flow through 5 mL of Pierce Iminobiotin Agarose (Thermo Scientific) in a gravity flow column (G-Biosciences) that had been rinsed with DI water and pre-equilibrated with IBAC binding buffer. The column was next washed with 20 column volumes of IBAC binding buffer, and the streptavidin was eluted from the column with 6 column volumes of elution buffer (20 mM KH_2_PO_4_, pH 2.2). The eluate was collected, dialyzed into PBS, and concentrated using a 10 kDa MWCO centrifugal filter (Millipore Sigma). The concentration of streptavidin was quantified by measuring the UV absorption at 280 nm.

### Expression and purification of 0304-3H3 antibody

The genes encoding the variable regions of the heavy chain and light chain of the 0304-3H3 antibody^21^ were cloned into the TGEX-HC and TGEX-LC vectors (Antibody Design Labs), respectively, by Gene Universal, Inc. (Newark, DE). The plasmids were co-transfected in a 2:1 light chain to heavy chain ratio into Expi293F cells (RRID: CVCL_D615) using the ExpiFectamine Transfection Kit (Thermo Fisher Scientific) and associated protocol. After a 4-day incubation, the culture was centrifuged at 5,500*×g* for 20 min. The supernatant was diluted in PBS and filtered before being purified by using a 1 mL MabSelect SuRe column (Cytiva) according to the manufacturer's protocol. The concentration of the purified 0304-3H3 antibody was quantified using the BCA assay (Thermo Scientific).

### In vitro biotinylation of AviTagged proteins

The BirA-500 kit (Avidity LLC) and general protocol were used to biotinylate the AviTagged MS2 and S2 proteins. In brief, the proteins were buffer exchanged into 20 mM Tris, 20 mM NaCl, pH 8.0. The concentration of protein in solution was adjusted to either 45 μM for MS2 or 15 μM for S2 and S2_mutS2’_ before adding the recommended amount of Biomix B (a proprietary mixture of biotin, ATP, and magnesium acetate). The recommended amount of BirA was added to the MS2 solution, while three times the recommended amount of BirA was added to the S2 solutions. These solutions were incubated at 37 °C for 2 h while shaking vigorously. After the 2-h incubation, the solutions were moved to a nutator at 4 °C for overnight incubation. Finally, the biotinylated proteins were separated from the biotinylation reagents using a Superdex 200 increase 10/300 column (Cytiva) and quantified by using the BCA assay (Thermo Scientific).

### Assembly of MS2-SA VLP

The assembly of MS2-SA VLP has been previously described.^37^ Approximately 1 mL of biotinylated MS2 at a concentration of about 0.7 mg/mL was added 2.5 μL at a time to stirred streptavidin that was in approximately 20-times molar excess and at a concentration of around 60 mg/mL. This mixture was stirred for 30 min at room temperature before the MS2-SA VLP was separated from excess streptavidin using a Superdex 200 increase 10/300 column (Cytiva). To quantify the purified MS2-SA VLP, a small sample of the MS2-SA VLP in Nu-PAGE lithium dodecyl sulfate (LDS) sample buffer (Invitrogen) was heated at 90 °C for at least 10 min and run on an SDS-PAGE gel with heated streptavidin standards of known mass.

### Assembly of VLP-S2 and VLP-S2_mutS2’_

MS2-SA and biotinylated S2 or S2_mutS2’_ were mixed in a ratio determined using analytical SEC. Mixtures consisting of 5 μg of S2 or S2_mutS2’_ and varying amounts of MS2-SA were run through a Superdex 200 increase 10/300 SEC column (Cytiva). The ratio of the mixture with the least amount of MS2-SA that resulted in a chromatogram without a peak corresponding to excess S2 or S2_mutS2’_ was the stoichiometric ratio used to generate VLP-S2 and VLP-S2_mutS2’_ for characterization and immunization.

### SDS-PAGE

Protein samples were diluted with 5 μL of Nu-PAGE lithium dodecyl sulfate (LDS) sample buffer (Invitrogen). These protein samples and PageRuler Plus Prestained Protein Ladder (Thermo Scientific) were loaded into the wells of a 4–12% Bis-Tris gel (Invitrogen). The gel was run in MES-SDS buffer at 110 V for 60 min while being chilled at 4 °C. The gel was stained with SimplyBlue SafeStain (Invitrogen), destained, and imaged using the ChemiDoc MP imaging system (Bio-Rad).

### Characterization of S2, S2_mutS2’_, VLP-S2, and VLP-S2_mutS2’_ by ELISA

Antigen (0.1 μg S2 and S2_mutS2’_ – alone and on VLP) was coated onto Nunc MaxiSorp 96-well flat-bottom plates (Invitrogen). The antigen solution was incubated for 1 h, before the wells were emptied and 5% BSA (Millipore) in PBST (PBS with 0.05% Tween-20) was added to the wells. This BSA solution remained in the wells for 45 min, after which it was discarded from the plate and each well was washed with 200 μL of PBST three times. Next, primary antibody (0304-3H3) was diluted in 1% BSA in PBST and a final volume of 100 μL was added to each well. The moles of antibody per well were equivalent to the moles of S2 trimer that had been coated in the well. The plate was left to incubate with the primary antibody for an hour, after which the plate was emptied, and each well was washed with 200 μL of PBST three times. Then 100 μL of the secondary antibody, horseradish peroxidase-conjugated anti-human IgG Fc fragment antibody (MP Biomedicals; SKU: 08674171, 1:5000 dilution) in 1% BSA in PBST, was added to each well. The secondary antibody solution remained in the plate for 1 h, after which the solution was discarded, and the wells of the plate were washed with 200 μL of PBST three times. The plate was then developed by adding 100 μL of TMB substrate solution (Millipore) to each well. The reaction was stopped after 3 min by adding 0.16 M sulfuric acid to each well. The absorbance of each well was then read at 450 nm using a Spectramax i3x plate reader (Molecular Devices).

### DLS

MS2-SA VLP was diluted in PBS to 100 μL such that there was 1 μg of SA in solution. VLP-S2 and VLP-S2_mutS2’_ were each diluted in PBS to 100 μL such that there was 5 μg of S2 in solution. Each 100 μL solution was then pipetted into a UVette (Eppendorf), which was inserted into a DynaPro NanoStar Dynamic Light Scattering detector (Wyatt Technology). Dynamics software (Wyatt Technology) brought the temperature of the measurement cell to 25 °C. The detector then proceeded with the measurement. Each measurement was the result of 10 acquisitions and was output as % Intensity, which could be converted to % Mass using the Isotropic Spheres model.

### Negative stain transmission electron microscopy

Conventional native-stain transmission electron microscopy (TEM) was performed, as described previously.^40^ Briefly, 4 μl of diluted samples were applied onto glow-discharged mesh copper grids (CF300-Cu; Electron Microscopy Science, PA), washed with PBS (1X), stained in droplets of 1% phosphotungstic acid (PTA, PH 6–7) for 1 min. The grids were then blotted from the grid backside and air-dried inside a Petri dish for at least 30 min under room temperature to minimize the negative-stain artifacts of flattening and stacking.^41^ The negative-stain grids were imaged in low-dose mode (50 e^−^/Å), using a Talos L120C transmission electron microscope (Thermo Fisher Scientific, previously FEI, Hillsboro, OR) at 120 kV. Images were acquired on a 4k x 4k Ceta CMOS camera microscope (Thermo Fisher Scientific), using SerialEM 3.8.^42^

### Plunge freezing and cryo-electron microscopy

4 μL of the VLP suspension was added to a glow discharged copper grid (C-Flat 1.2/1.3, 400mesh, Protochips) with an extra layer of carbon (∼2 nm) on the holey carbon surface. Grids were plunge frozen using a Vitrobot Mark IV (ThermoScientific) and stored in liquid nitrogen until imaging. Cryo-electron microscopy (cryo-EM) imaging was performed on a Titan Krios (ThermoScientific Hillsboro, OR, USA) operated at 300 kV. Images (defocus of −2–5 μm) were recorded on a post-GIF Gatan K3 camera in EFTEM mode (2.176 Å/pixel) with a 20-eV slit, CDS counting mode, using SerialEM 3.8.^42^ A total dose of 30–40 e/Å^2^ was used and 40 frames were saved (∼1.2 e/Å^2^ per frame). Frames were motion-corrected in MotionCor2.^43^ Images were low pass filtered to 10 Å^2^ for better visualization and contrast using EMAN2.^44^

### Cells and Viruses

Vero E6/TMPRSS2 cells obtained from the National Institute of Infectious Diseases, Japan^45^ were maintained in high glucose Dulbecco's modified Eagle's medium (DMEM) containing 10% fetal bovine serum (FBS) and antibiotic/antimycotic solution along with G418 (1 mg/mL). Virus stocks were propagated and tittered on Vero E6 TMPRSS2 cells. The following challenge viruses were used in the hamster studies: SARS-CoV-2/UT-NCGM02/Human/2020/Tokyo (NCGM02), hCoV-19/USA/WI-UW-5250/2021 (B.1.617.2, delta), hCoV-19/USA/MD-HP01542/2021 (B.1.351, beta), hCoV-19/USA/WI-WSLH-221686/2021 (B.1.1.529 BA.1, Omicron), and Pg CoV, BetaCoV/pangolin/Guandong/1/2019.

### Animal studies

Animals were housed for five days before the start of the study in rooms with controlled temperature and humidity along with a 12-h light and dark cycle. Food and water were available *ad libitum* along with enrichment. Animals were monitored at least twice daily by trained personnel. Animals were randomly assigned to infection groups and researchers were not blinded on the selection of animals. Samples sizes of three or four hamsters and four to five mice were determined based on prior *in vivo* virus challenge experiments; no sample size calculations were performed to power each study. No animals were excluded, and all data was included in the analysis.

Wild-type Syrian hamsters (females; 4–5 weeks old; Envigo) were immunized with 20 μg of SARS-CoV-2 S2 protein presented on VLPs, a mutant S2 protein presented on VLPs, or VLPs without the S2 protein, by subcutaneous inoculation. One of the following adjuvants were added to each vaccine preparation before inoculation: AddaVax (InvivoGen; equal volume vaccine and adjuvant), QS-21 (Desert King; 25 μg), R848 (InvivoGen, 25 μg), or AddaS03 + pIC (InvivoGen equal volume of AddaS03 plus 100 μg of pIC). Animals were infected by intranasal inoculation with 10^3^ plaque-forming units (pfu) of SARS-CoV-2 while under isoflurane anesthesia. Three days after infection, animals were humanely sacrificed by overdose of isoflurane, and lung tissue and nasal turbinate samples were collected to measure the amount of virus.

Wild-type BALB/c mice (females; 8–10 weeks old; Taconic Biosciences) were immunized by subcutaneous inoculation with 14 μg of SARS-CoV-2 S2 protein presented on VLPs or VLPs without the S2 protein, adjuvanted with AddaS03 + pIC. Four weeks after a single immunization, serum was collected from a group of animals, while another group of animals were infected by intranasal inoculation with 2 × 10^3^ pfu of mouse-adapted SARS-CoV-2^46^ while under isoflurane anesthesia. Three days after infection, animals were humanely sacrificed by overdose of isoflurane, and lung tissue samples were collected to measure the amount of virus.

Virus titers in the tissues were determined on confluent Vero E6/TMPRSS2 cells by infecting cells with 100 μL of undiluted or 10-fold dilutions (10^−1^–10^−5^) of clarified lung and nasal turbinate homogenates. After a 30-min incubation, the inoculum was removed, the cells were washed once, and then overlaid with 1% methylcellulose solution in DMEM with 5% FBS. The plates were incubated for three days, and then the cells were fixed and stained with 20% methanol and crystal violet in order to count the plaques.

### Detection of antibodies against the SARS-CoV-2 S2 in immunized hamsters

ELISAs were performed using recombinant spike SARS-CoV-2 proteins either produced in Expi293F cells (RRID: CVCL_D615) and then C-terminal His-tag purified by using TALON metal affinity resin (Wuhan, B.1.351, B.1.617.2, B.1.1.529 BA.1 and BA.2, RsSCH014, and Pg-CoV spike antigens) or purchased from Sino Biological (229E, OC43, HKU-1, NL63, and CoV-1 strain Tor2 spike antigens). ELISA plates were coated overnight at 4 °C with 50 μL of spike antigen at a concentration of 2 μg/mL in PBS. After blocking with PBS containing 0.1% Tween 20 (PBS-T) and 3% milk powder, the plates were incubated in duplicate with heat-inactivated serum diluted in PBS-T with 1% milk powder. A hamster IgG secondary antibody conjugated with horseradish peroxidase (1:7000 dilution, ThermoFisher; RRID:AB_2535672) was used for detection. Plates were developed with SigmaFast o-phenylenediamine dihydrochloride solution (Sigma), and the reaction was stopped with the addition of 3M hydrochloric acid. The absorbance was measured at a wavelength of 490 nm (OD_490_). Background absorbance measurements from serum collected before immunization was subtracted from serum collected before challenge for each dilution. IgG antibody endpoint titers were defined as the highest serum dilution with an OD_490_ cut-off value of 0.15.

### Focus reduction neutralization test (FRNT)

Neutralization of SARS-CoV-2 was characterized by using a focus reduction neutralization test. Serial dilutions of serum from vaccinated hamsters starting at a final concentration of 1:20 were mixed with ∼1500 focus-forming units (FFU) of virus/well and incubated for 1 h at 37 °C. Pooled serum from hamsters vaccinated with VLP without the S2 protein served as a control. The antibody-virus mixture was inoculated onto Vero E6/TMPRSS2 cells in 96-well plates and incubated for 1 h at 37 °C. An equal volume of methylcellulose solution was added to each well. The cells were incubated for 16 h at 37 °C and then fixed with formalin. After the formalin was removed, the cells were immunostained with a mouse monoclonal antibody against SARS-CoV-1/2 nucleoprotein [clone 1C7C7 (Sigma–Aldrich; RRID:AB_2893327)], followed by a horseradish peroxidase-labeled goat anti-mouse immunoglobulin (SeraCare Life Sciences). The infected cells were stained with TrueBlue Substrate (SeraCare Life Sciences) and then washed with distilled water. After cell drying, the focus numbers were quantified by using an ImmunoSpot S6 Analyzer, ImmunoCapture software, and BioSpot software (Cellular Technology). The results are expressed as the 50% focus reduction neutralization titer (FRNT_50_). The FRNT_50_ values were calculated by using Prism 9 (Graphpad Software). Percent neutralization was calculated as 100 × (1 – [ratio of foci in the presence of sera from hamsters vaccinated with VLP-S2_mutS2’_ and foci in the presence of pooled sera from hamsters vaccinated with VLP control]). The FRNT_50_ value was then calculated from the normalized percent neutralization using a four-parameter nonlinear regression in Graphpad Prism. A similar approach was used to characterize sera from immunized mice.

### Biosafety statement

Research with SARS-CoV-2 and related SARS-like viruses was performed under biosafety level 3 agriculture (BSL-3Ag) containment at the Influenza Research Institute with an approved protocol reviewed and approved by the University of Wisconsin–Madison's Institutional Biosafety Committee. The laboratory is designed to meet and exceed the standards outlined in *Biosafety in Microbiological and Biomedical Laboratories* (6th edition).

### Statistics

**Fig. 2:**
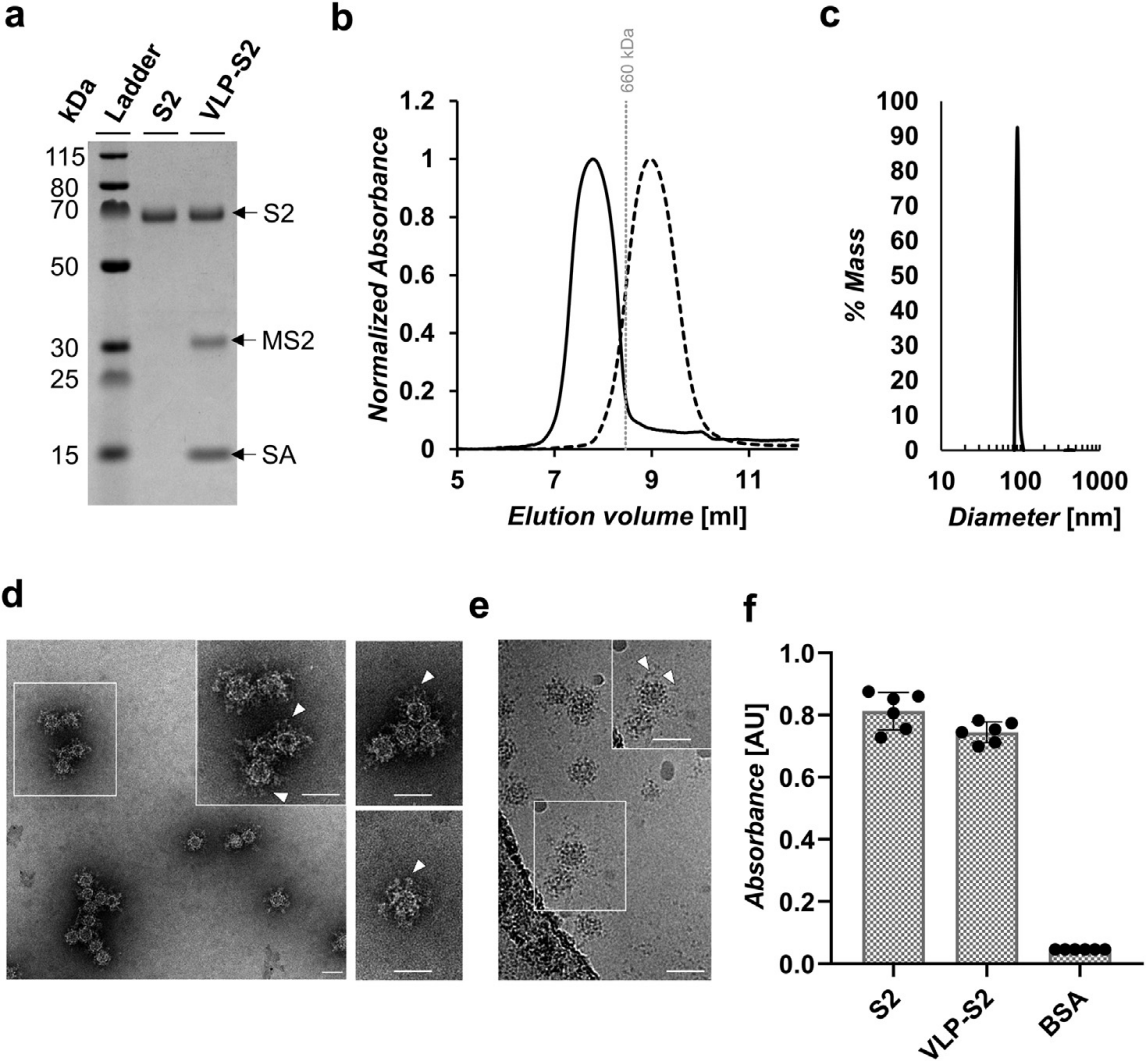
**Characterization of S2 and VLP-S2.(a)** SDS-PAGE characterization of S2 and VLP-S2. S2 was deglycosylated with PNGase F. The samples were heated with β-mercaptoethanol and LDS sample buffer. The unprocessed gel is shown in Fig. S2a. **(b)** Size exclusion chromatography traces for S2 (dashed line) and VLP-S2 (solid line). The vertical gray line represents the peak elution volume of the molecular weight standard thyroglobulin (660 kDa). The column void volume is 7.2 mL. **(c)** Characterization of the VLP-S2 by dynamic light scattering. **(d)** Negative-stain transmission electron micrograph of VLP-S2. Arrowheads (white) indicate the S2 protein on the VLP surface. Scale bars = 50 nm. **(e)** Cryo-EM of vitrified VLP-S2. Arrowheads (white) indicate the S2 protein on the VLP surface. Scale bars = 50 nm. **(f)** Characterization of the binding of anti-S2 antibody 0304-3H3 to S2 and VLP-S2 by ELISA. (mean ± SD, n = 6: two independent assays, each with three technical replicates).

**Fig. 3:**
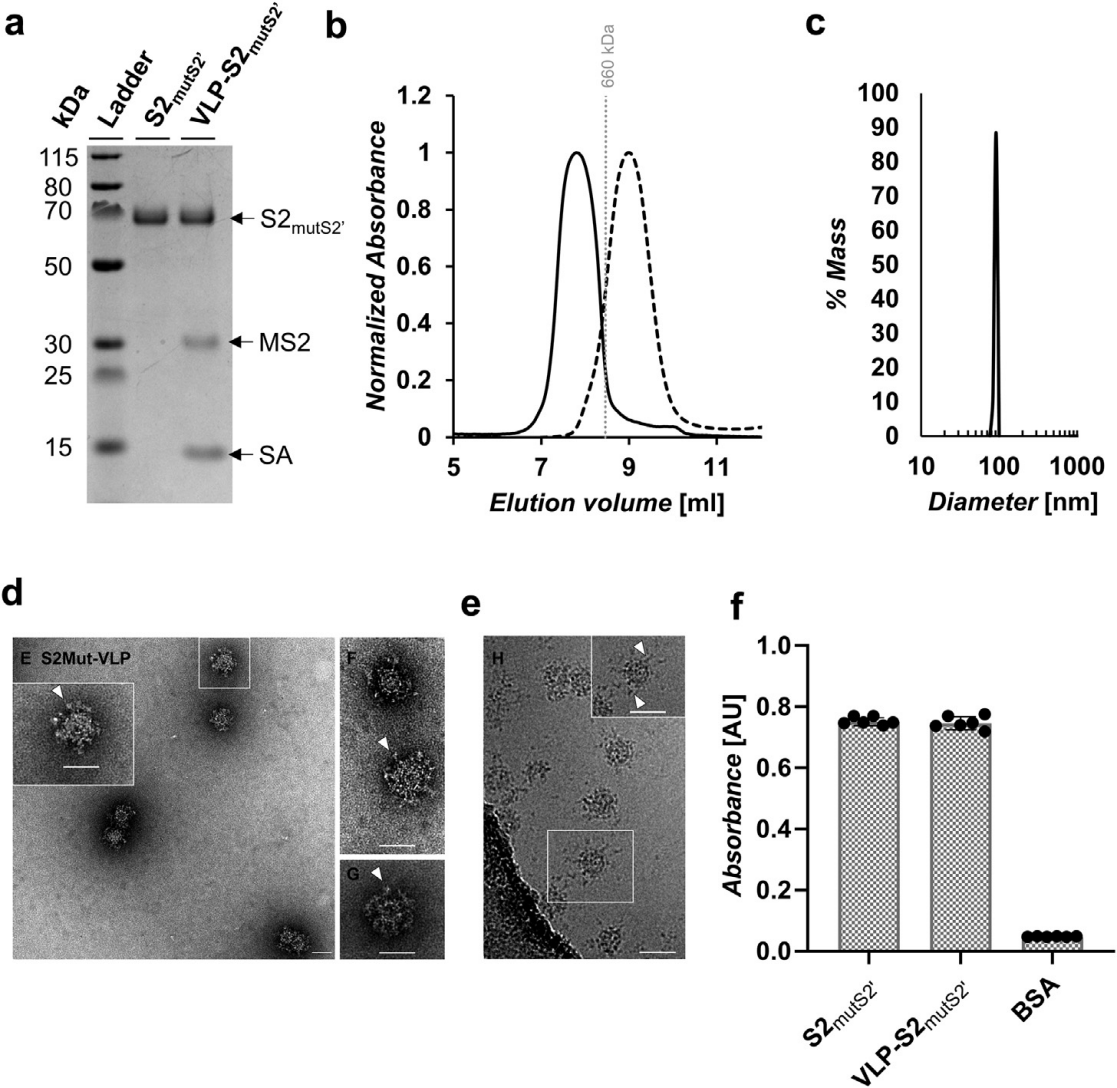
**Characterization of S2_mutS2’_ and VLP-S2_mutS2’_.(a)** SDS-PAGE characterization of S2_mutS2’_ and VLP-S2_mutS2’_. S2_mutS2’_ was deglycosylated with PNGase F. The samples were heated with β-mercaptoethanol and LDS sample buffer. The unprocessed gel is shown in Fig. S2a. **(b)** Size exclusion chromatography traces for S2_mutS2’_ (dashed line) and VLP-S2_mutS2’_ (solid line). The vertical gray line represents the peak elution volume of the molecular weight standard thyroglobulin (660 kDa). The column void volume is 7.2 mL. **(c)** Characterization of the VLP-S2_mutS2’_ by dynamic light scattering. **(d)** Negative-stain transmission electron micrograph of VLP-S2_mutS2’_. Arrowheads (white) indicate the S2 _mutS2’_ protein on the VLP surface. Scale bars = 50 nm. **(e)** Cryo-EM of vitrified VLP-S2_mutS2’_. Arrowheads (white) indicate the S2 _mutS2’_ protein on the VLP surface. Scale bars = 50 nm. **(f)** Characterization of the binding of anti-S2 antibody 0304-3H3 to S2 and VLP-S2 by ELISA. (mean ± SD, n = 6: two independent assays, each with three technical replicates).

**Fig. 4:**
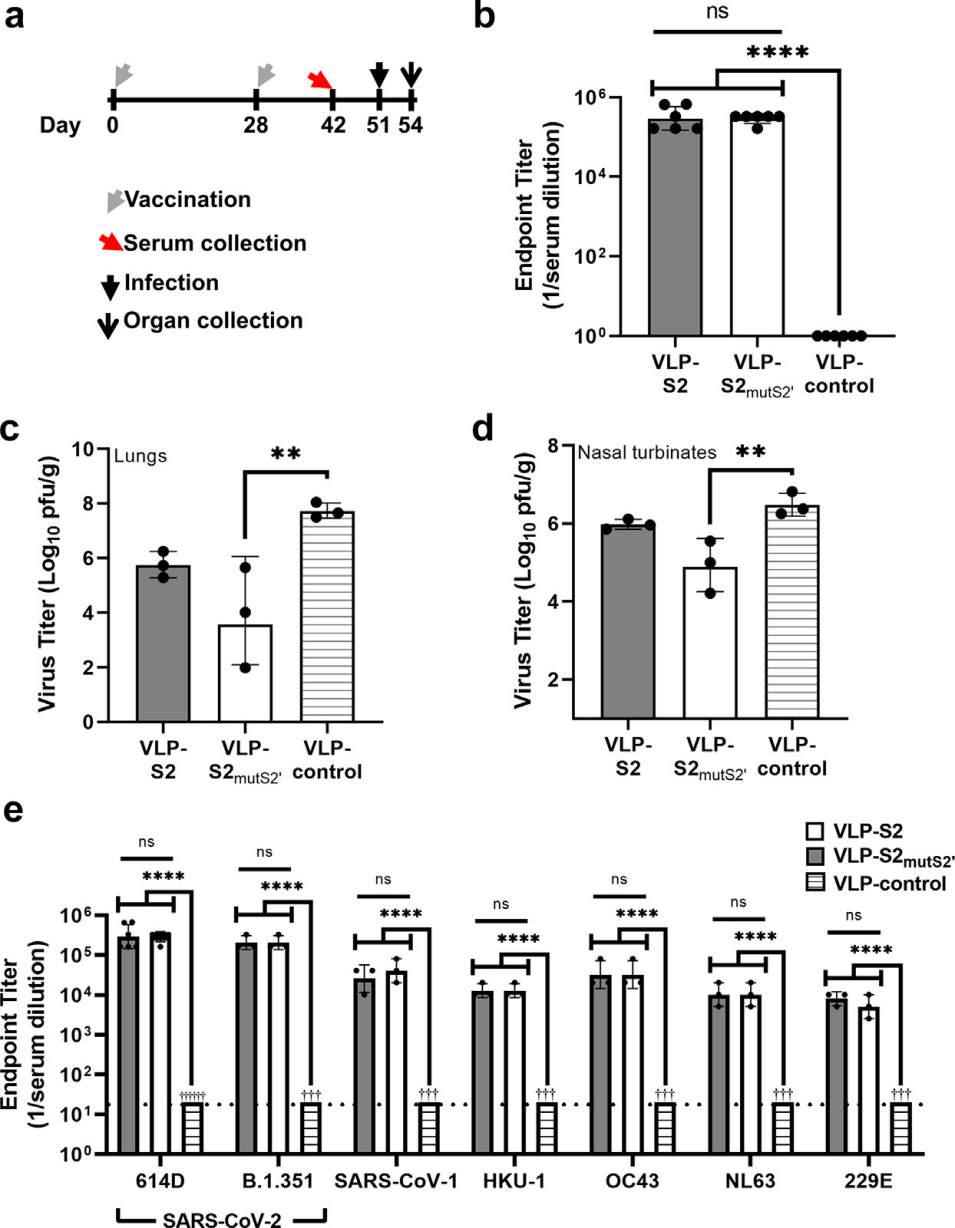
**Characterization of the efficacy of VLP-S2 and VLP-S2_mutS2’_ in hamsters.(a)** Schedule for hamster vaccination, serum collection, infection with SARS-CoV-2, and organ collection. **(b)** Antibody endpoint titers of sera from hamsters immunized with either VLP-S2, VLP-S2_mutS2_, or VLP-control against SARS-CoV-2 spike protein (geometric mean with geometric SD, n = 6, biological replicates = 3: two independent assays with sera from 3 hamsters). ns: not statistically significant, ∗∗∗∗*P* < 0.0001 [one-way analysis of variance (ANOVA) and Tukey post-hoc multiple comparison between groups (α = 0.05)]. **(c)** Viral titer in the lungs of hamsters immunized with either VLP-S2, or VLP-S2_mutS2’_, or VLP-control three days after infection with SARS-CoV-2 (mean with SD, n = 3, biological replicates = 3: tissues from 3 hamsters). ∗∗*P* = 0.0098 [one-way analysis of variance (ANOVA) and Dunnett post-hoc multiple comparison between groups (α = 0.05)]. **(d)** Viral titer in the nasal turbinates of hamsters immunized with either VLP-S2, or VLP-S2_mutS2_, or VLP-control three days after SARS-CoV-2 infection (mean with SD, n = 3, biological replicates = 3: tissues from 3 hamsters). ∗∗*P* = 0.0057 [one-way analysis of variance (ANOVA) and Dunnett post-hoc multiple comparison between groups (α = 0.05)]. **(e)** Antibody endpoint titers of sera from hamsters immunized with either VLP-control, VLP-S2 (gray), or VLP-S2_mutS2_ (white) against spike proteins of the original Wuhan-Hu-1 SARS-CoV-2 (614D), the SARS-CoV-2 variant B.1.351, SARS-CoV-1, and the four endemic human coronaviruses HKU-1, OC43, NL63, and 229E (geometric mean with geometric SD, n = 6, biological replicates = 3 against SARS-CoV-2 614D S protein: two independent assays with sera from 3 hamsters; n = 3, biological replicates = 3 against all other S proteins: sera from 3 hamsters). ns: not statistically significant, ∗∗∗∗*P* < 0.0001 [one-way analysis of variance (ANOVA) and Tukey post-hoc multiple comparison between groups (α = 0.05)].

**Fig. 5:**
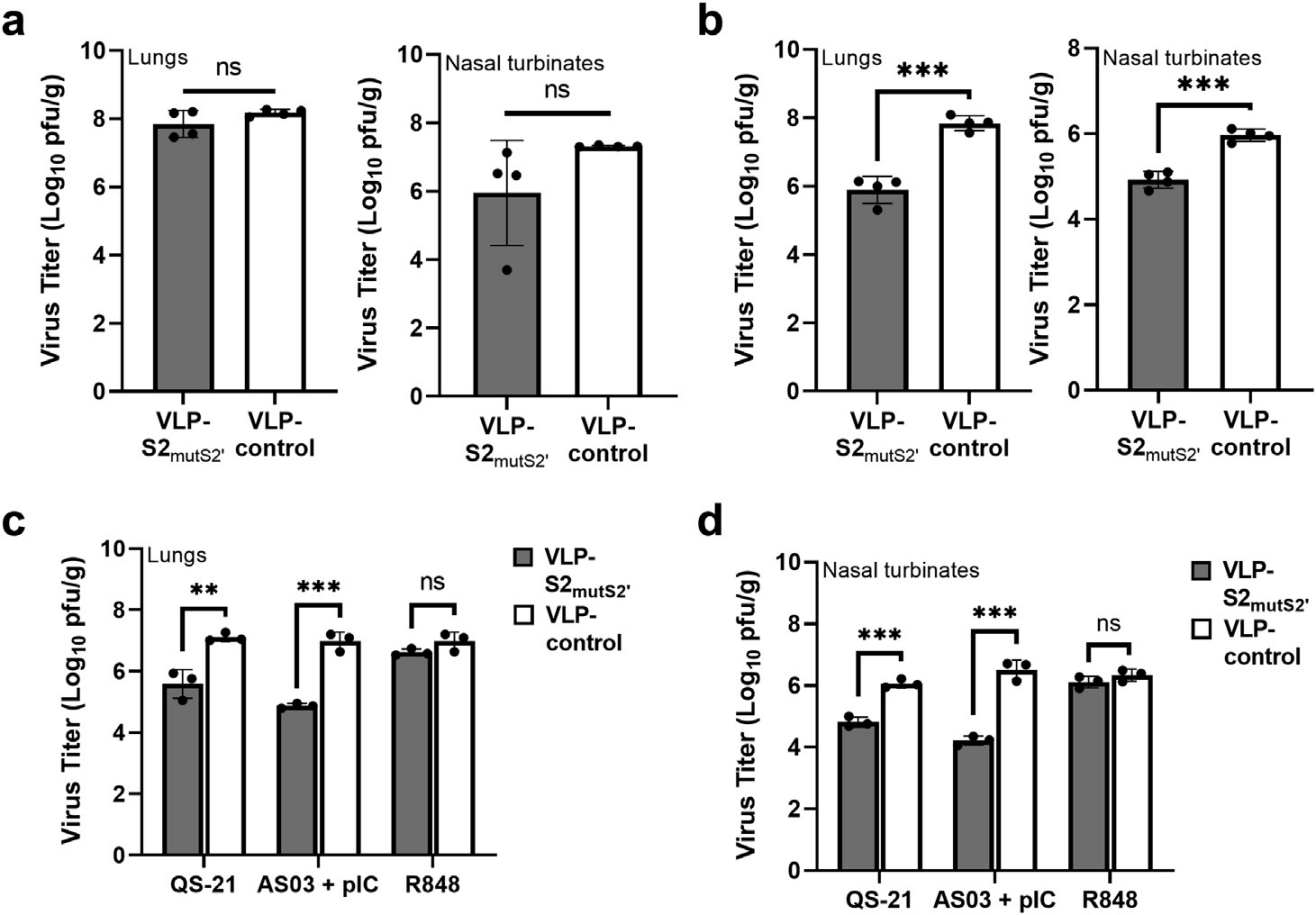
**Optimization of the efficacy of VLP-S2_mutS2’_ in hamsters.(a)** Viral titers in the lungs (left) and nasal turbinates (right) of hamsters immunized with two doses of either VLP-S2_mutS2’_ or VLP-control adjuvanted with AddaVax, three days after infection with B.1.617.2 (mean with SD, n = 4, biological replicates = 4: tissues from four hamsters). ns: not statistically significant [two-tailed Welch's t-test]. **(b)** Viral titers in the lungs (left) and nasal turbinates (right) of hamsters immunized with three doses of either VLP-S2_mutS2’_ or VLP-control adjuvanted with AddaVax, three days after infection with B.1.617.2 (mean with SD, n = 3, biological replicates = 3: tissues from three hamsters). ∗∗∗*P* = 0.0001 for lungs and nasal turbinates [two-tailed unpaired t-test]. **(c)** Viral titers in the lungs of hamsters immunized with two doses of either VLP-S2_mutS2’_ or VLP-control mixed with various adjuvants, three days after infection with B.1.617.2 (mean with SD, n = 3, biological replicates = 3: tissues from three hamsters). ns: not statistically significant, ∗∗*P* = 0.0056 for QS-21, ∗∗∗*P* = 0.0003 for AS03 + pIC, p = 0.1243 for R848 [two-tailed unpaired t-test]. **(d)** Viral titers in the nasal turbinates of hamsters immunized with two doses of either VLP-S2_mutS2’_ or VLP-control mixed with various adjuvants, three days after infection with B.1.617.2 (mean with SD, n = 3, biological replicates = 3: tissues from three hamsters). ns: not statistically significant, ∗∗∗*P* = 0.0006 for QS-21, ∗∗∗*P* = 0.0003 for AS03 + pIC, p = 0.2181 for R848 [two-tailed unpaired t-test].

**Fig. 6:**
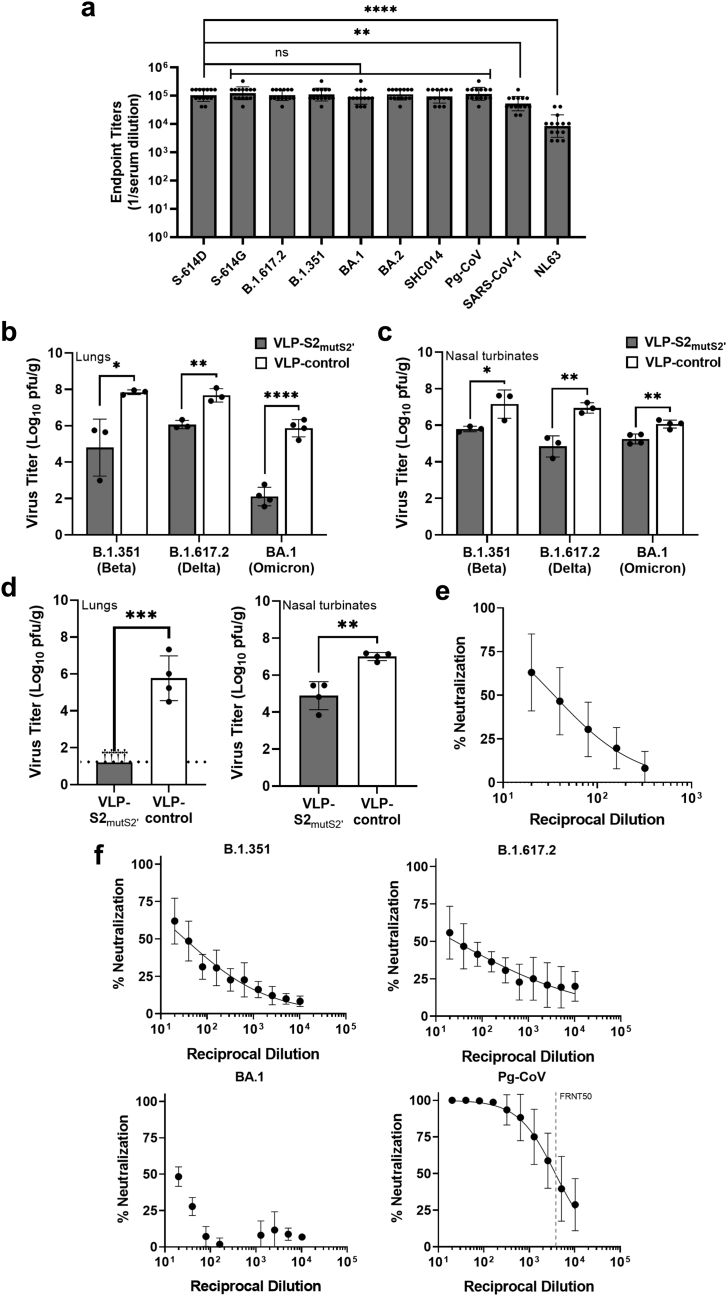
**Evaluating the breadth of the efficacy of the optimal immunization regimen in hamsters via a challenge with SARS-CoV-2 variants of concern and pangolin coronaviruses.(a)** Viral antibody endpoint titers against S proteins from various coronaviruses (geometric mean with geometric SD, n = 14, biological replicates = 14: sera from 14 hamsters) ns: not statistically significant, ∗∗*P* = 0.015 for SARS-CoV-1, ∗∗∗∗*P* < 0.0001 for NL63 [Brown–Forsythe and Welch ANOVA tests]. **(b)** Viral titers in the lungs of hamsters immunized with either VLP-S2_mutS2’_ or VLP-control adjuvanted with AS03 + pIC, three days after infection with SARS-CoV-2 variants (mean with SD, n = 4, biological replicates = 4 for BA.1: sera from four hamsters, n = 3, biological replicates = 3 for all other coronaviruses: tissues from three hamsters). ∗*P* = 0.0284 for Beta, ∗∗*P* = 0.003 for Delta, ∗∗∗∗*P* < 0.0001 for Omicron [two-tailed Welch's t-test]. **(c)** Viral titers in the nasal turbinates of hamsters immunized with either VLP-S2_mutS2’_ or VLP-control adjuvanted with AS03 + pIC, three days after infection with SARS-CoV-2 variants (mean with SD, n = 4, biological replicates = 4 for BA.1: tissues from four hamsters, n = 3, biological replicates = 3 for all other variants: tissues from three hamsters). ns: not statistically significant, ∗*P* = 0.042 for Beta, ∗∗*P* = 0.0048 for Delta, ∗∗*P* = 0.0034 for Omicron [two-tailed Welch's t-test]. **(d)** Viral titers in the lungs (left) and nasal turbinates (right) of hamsters immunized with either VLP-_S2mutS2’_ or VLP-control adjuvanted with AS03 + pIC, three days after infection with Pg-CoV (mean with SD, n = 4, biological replicates = 4: tissues from four hamsters) ∗∗*P* = 0.0017, ∗∗∗*P* = 0.0002 [two-tailed Welch's t-test]. † - No infectious virus was detected in the lungs of immunized hamsters. Detection limit (dotted line) = 1.3 log_10_ pfu/g. **(e)** Percent neutralization against SARS-CoV-2 early isolate S-614G after immunization with VLP-S2_mutS2’_. (mean with SD, n = 14, biological replicates = 14 for VLP-S2_mutS2’_: sera from 14 hamsters). **(f)** Percent neutralization against B.1.351, B.1.617.2, BA.1, and Pg-CoV after immunization with VLP-S2_mutS2’_. FRNT_50_ = 3732 for Pg-CoV. (mean with SD, n = 6, biological replicates = 6: sera from 6 hamsters).

**Fig. 7:**
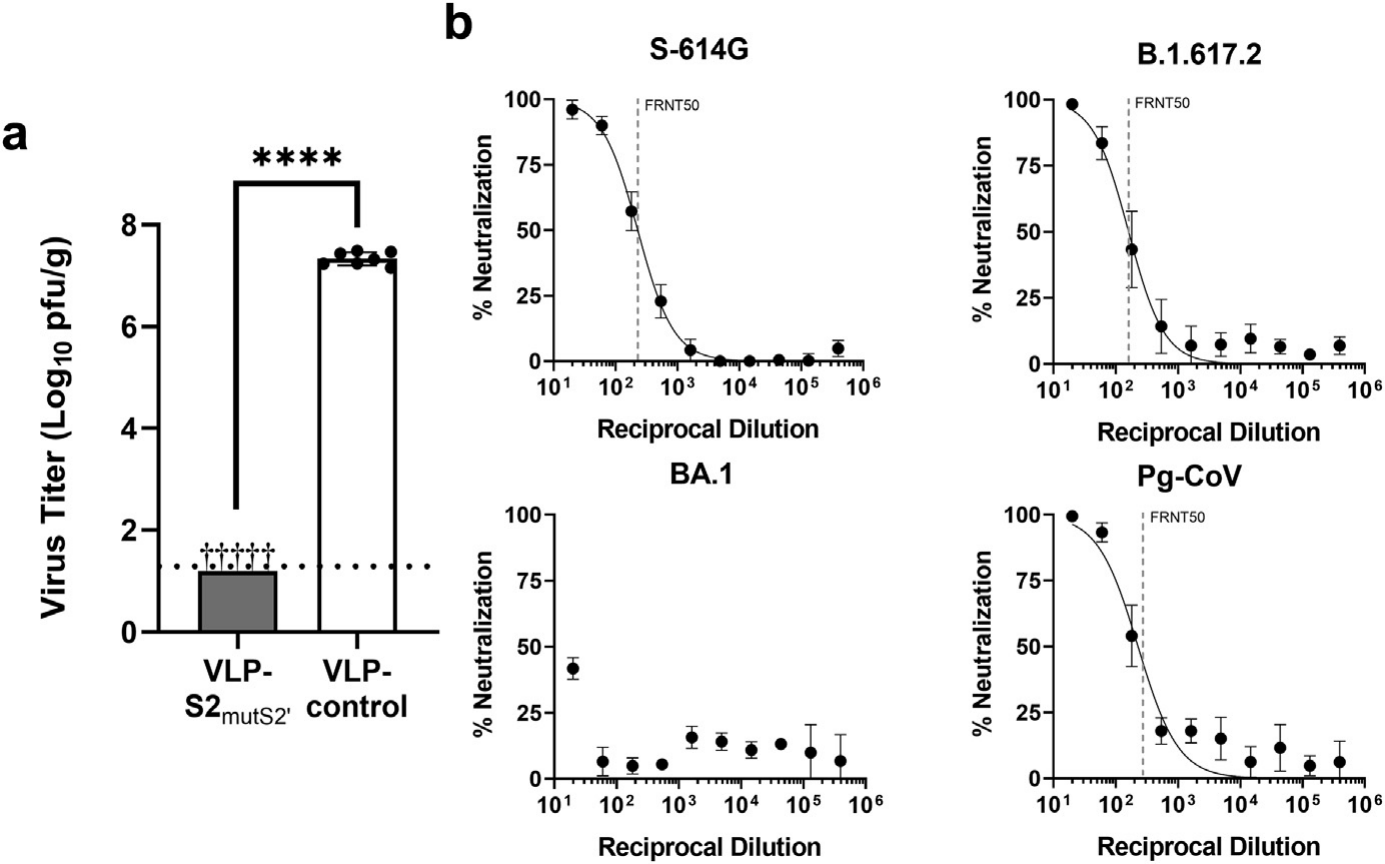
**Evaluating the efficacy of VLP-S2_mutS2’_ in a mouse model.(a)** Viral titers in the lungs of mice immunized with one dose of either VLP-S2_mutS2’_ or VLP-control adjuvanted with AS03 + pIC, three days after infection with mouse-adapted SARS-CoV-2 strain, MA10. (mean with SD, biological replicates and n = 5 for VLP-S2_mutS2’_: tissues from five mice, biological replicates and n = 7 for VLP-control: tissues from seven mice). ∗∗∗∗*P* < 0.0001 [two-tailed Welch's t-test]. † - No infectious virus was detected in the lungs of immunized mice. Detection limit (dotted line) = 1.3 log_10_ pfu/g. **(b)** Percent neutralization against SARS-CoV-2 early isolate S-614G, B.1.617.2, BA.1, and Pg-CoV after immunization with one dose of VLP-S2_mutS2’_. FRNT50 = 228 for S-614G, 161 for B.1.617.2, <20 for BA.1, and 228 for Pg-CoV (mean with SD, biological replicates and n = 3 for BA.1: sera from three mice, biological replicates and n = 4 for other coronaviruses: sera from four mice).

*In vitro* characterizations of the binding of 0304-3H3 to VLP-S2 and VLP-S2_mutS2’_ using ELISA (Figs. 2e and 3e) were each conducted twice independently with three technical replicates for each condition. The data are presented as the mean ± SD. For *in vivo* characterization of VLP-S2 and VLP-S2_mutS2’_, there were three groups (receiving either VLP-S2, VLP-S2_mutS2’_, or VLP-control) each with three hamsters (n = 3). To determine the resulting endpoint titers against the SARS-CoV-2 spike protein (Fig. 4b; Table 1), two independent assays were conducted using sera from each hamster. Significance was determined by a one-way analysis of variance (ANOVA) and Tukey post-hoc multiple comparison between groups (α = 0.05). For further *in vivo* characterization of VLP-S2_mutS2’_, studies were conducted with two groups each (receiving either VLP-S2_mutS2’_ or VLP-control). Endpoint titers (Fig. 4e; Fig. 6a) were determined by conducting a single assay using sera from each hamster (3 hamsters for Fig. 4e; 14 hamsters for Fig. 6a). The data are presented as the geometric mean with the geometric SD factor and significance was determined by a one-way analysis of variance (ANOVA) and Tukey post-hoc multiple comparison between groups (α = 0.05). Viral titers in the lungs and nasal turbinates of hamsters immunized with either VLP-S2, VLP-S2_mutS2’_, or VLP-control 3 days after SARS-CoV-2 infection (Fig. 4c and d) were presented as the mean with SD (n = 3) and the significance was determined by a one-way analysis of variance (ANOVA) and Dunnett post-hoc multiple comparison between groups (α = 0.05). For all tests of significance, assumptions of the normality of residuals and homogeneity of variance were validated by the D'Agostino-Pearson test and the Brown–Forsythe test, respectively. Viral titers in the lungs and nasal turbinates of hamsters immunized with either VLP-S2_mutS2’_ or VLP-control 3 days after infection with a SARS-CoV-2 variants or Pg-CoV (Fig. 5, Fig. 6a–d) were presented as the mean with SD (n = 3 or 4) and the significance was determined by either unpaired t-test or Welch's t-test. Three hamsters per group were used for the study presented in Fig. 5a and b and four hamsters per group for the study in Fig. 5c and d. For the study in Fig. 6, three hamsters per group were challenged with either B.1.351 or B.1.617.2, and four hamsters per group were challenged with either BA.1 or Pang-CoV. For all measurements of viral titers, tissue samples from each hamster were used in a single assay. For all tests of significance, assumption of the normality of residuals was validated by the Shapiro–Wilk and Kolmogorov–Smirnov tests. The homogeneity of variances was determined by the F-test of equality of variances. For the study presented in Fig. 7, five mice were immunized with VLP-S2_mutS2’_ and seven mice were immunized with VLP-control. For the measurement of lung viral titers, a single assay was conducted using tissue samples from each mouse. Lung viral titers were presented as the mean with SD (n = 5 or 7) and the significance was determined by Welch's t-test. Percent neutralization in Fig. 6, Fig. 7e and f and 7b were presented as mean with SD. A single assay was conducted using sera from each animal (n = 14 hamsters for Fig. 6e, n = 6 hamsters for Fig. 6f, n = 3 mice for BA.1 in Fig. 7b, and n = 4 mice for other coronaviruses in Fig. 7b). All statistical analysis was carried out using Prism 9 (GraphPad).

**Table 1:** Antibody responses to VLP-S2 and VLP-S2_mutS2’_ after prime and boost in Syrian hamsters.

	Spike IgG Endpoint Titer													
	SARS-CoV-2													
	614D		B.1.351		SARS-CoV-1		HKU-1		OC43		NL63		229E	
Vaccine group	Geometric mean	Geometric SD factor	Geometric Mean	Geometric SD factor	Geometric mean	Geometric SD factor	Geometric mean	Geometric SD factor	Geometric mean	Geometric SD factor	Geometric mean	Geometric SD factor	Geometric mean	Geometric SD factor
MS2-SA VLP	<20	–	<20	–	<20	–	<20	–	<20	–	<20	–	<20	–
VLP-S2	292,667	1.98	206,425	1.49	25,803	2.23	12,902	1.49	32,404	2.23	10,240	2.00	8127	1.49
VLP-S2_mutS2'_	291,930	1.33	206,425	1.49	40,960	2.00	12,902	1.49	32,510	2.23	10,240	2.00	5120	2.00

Viral antibody endpoint titers against the SARS-CoV-2 spike (three animals in each group). Endpoint titers using 2-fold diluted sera were expressed as the reciprocal of the highest dilution with an optical density at 490 nm cutoff value > 0.15; sera were collected on day 42 after the initial immunization.

### Ethics

These animal studies were approved by the Institutional Animal Care and Use Committee at the University of Wisconsin.

### Role of the funders

The funders of the study had no role in study design, data collection, data analysis, data interpretation, or writing of the report.

## Results

### Generation and *in vitro* characterization of S2 nanoparticle-based vaccines

We previously developed streptavidin-coated VLPs that we used to display biotinylated protein antigens such as the SARS-CoV-2 spike protein and DIII of the Zika virus envelope protein.^37^^,^^47^ In this study we have used these same VLPs to display the S2 subunit of the spike protein (Fig. 1b). The VLPs are based on the bacteriophage MS2 coat protein^48^; 90 MS2 coat protein homodimers self-assemble into an icosahedral structure.^49^ We used BL21(DE3) *E. coli* (*E. Coli*) to express a single chain dimer of the MS2 coat protein with an AviTag inserted in a surface loop that had been shown to tolerate peptide insertions (Table S1).^50^ The inserted AviTag allowed for site-specific biotinylation of each coat protein dimer. After expression, the VLPs were purified using Capto Core 700 resin and size exclusion chromatography (SEC). The VLPs were then biotinylated and subsequently separated from the biotinylation reagents using SEC. The biotinylated MS2 VLPs were added dropwise to a large excess of stirred streptavidin (SA), which had been expressed as inclusion bodies, refolded, and purified using iminobiotin affinity chromatography.^37^^,^^47^ The resulting MS2-SA VLPs were separated from excess streptavidin using size exclusion chromatography. Consistent with prior characterization,^37^ SDS-PAGE analysis of the MS2-SA VLPs indicated that there were approximately 70 streptavidin molecules bound to each MS2 biotin VLP (Fig. S1a). In addition, the MS2-SA VLPs were found to be pure and homogenous in size based on characterization by SEC (Fig. 1c), dynamic light scattering (DLS; Fig. 1d), negative-stain transmission electron microscopy (NS-TEM; Fig. 1e) and cryo-electron microscopy (cryo-EM; Fig. 1f).

Biotinylated S2 was next produced such that it could be displayed on the MS2-SA VLPs. We used Expi293F mammalian cells to express the HexaPro^36^ variant of the SARS-CoV-2 spike protein's S2 subunit with an N-terminal signal peptide, a C-terminal trimerization domain to promote stability, a C-terminal AviTag for biotinylation, and a C-terminal his-tag for purification (Table S1). The HexaPro variant contains 6 stabilizing proline mutations, (F817P, A892P, A899P, A942P, K968P, and V969P), as reported by Hsieh et al.^36^ The expressed S2 was purified using immobilized metal affinity chromatography (IMAC) and was then biotinylated *in vitro*. The biotinylated S2 was separated from biotinylation reagents using size exclusion chromatography and could then be displayed on the MS2-SA VLPs.

To determine the appropriate ratio of S2 to add to MS2-SA VLPs, analytical SEC was used. Mixtures of the two proteins were made that contained a constant amount of S2 and varying amounts of MS2-SA VLPs. The ratio of the mixture with the least amount of MS2-SA VLPs that displayed no indication of excess S2 in an SEC chromatogram was determined to be the approximate stoichiometric ratio. Further analysis by SDS-PAGE indicated that this stoichiometric ratio resulted in approximately 30 S2 molecules conjugated to each MS2-SA VLP (Fig. S1b). The MS2-SA and biotinylated S2 were mixed in this ratio to create the VLP-S2 immunogen.

The VLP-S2 immunogen was characterized *in vitro* using several different bioanalytical techniques. First, the proteins that made up VLP-S2 were characterized by SDS polyacrylamide gel electrophoresis (SDS-PAGE), which indicated that the proteins were pure (Fig. 2a). In addition, comparison of the molecular weight ladder to the bands representing deglycosylated S2 (∼63 kDa), biotinylated MS2 (∼29 kDa), and monomeric streptavidin (∼15 kDa) demonstrated that these proteins aligned as expected with molecular weight standards. The VLP-S2 was also analyzed using analytical SEC, where chromatograms were generated for VLP-S2, S2 alone, and the molecular weight standard thyroglobulin (Fig. 2b). The resulting UV trace corresponding to the VLP-S2 contained a single peak that appeared before the peak for S2 alone. Therefore, the VLP-S2 was free of excess S2 and was generally uniform in size. To obtain a direct size measurement of the VLP-S2, we used Dynamic Light Scattering (DLS) (Fig. 2c), NS-TEM (Fig. 2d), and cryo-EM (Fig. 2e). The DLS measurements indicated that the VLP-S2 construct was approximately 90 nm in diameter. Characterization of the VLP-S2 by NS-TEM and cryo-EM confirmed the presence and coating of the S2 protein on the surface of the MS2-SA VLP. NS-TEM analysis suggested that VLP-S2 was ∼65 nm in diameter on average (n = 300). The larger size indicated by DLS may be a result of the fact that scattering intensity is proportional to the sixth power of the radius, giving rise to a disproportionately higher weighting of larger particles. We next used ELISA to probe the binding of the anti-S2 monoclonal antibody 0304-3H3^21^ to S2 and VLP-S2 (Fig. 2f). This antibody bound to both the S2 and VLP-S2, suggesting that S2 retained its antigenicity after conjugation to VLPs.

In addition to the VLP-S2, we generated VLP-S2_mutS2’_ particles. The VLP-S2_mutS2’_ displayed an S2 variant (S2_mutS2’_) that contained S2′ cut site residues that had been mutated to glycine residues (Table S1). The purpose of this mutation was to prevent potential proteolytic cleavage of the S2 immunogen at the S2′ cut site. The VLP-S2_mutS2’_ was generated and characterized using the same procedures described above for the VLP-S2 (Fig. 3).

### Multivalent S2-based vaccines reduce coronavirus titers in the respiratory tissues

We next assessed the efficacy of the VLP-S2 and VLP-S2_mutS2’_ against an early isolate of SARS-CoV-2, SARS-CoV-2/UT-NCGM02/Human/2020/Tokyo (NCGM02; spike protein with aspartate (D) at position 614^45^). Hamsters were immunized with either VLP-S2, VLP-S2_mutS2’_, or MS2-SA VLP (VLP-control) alone, adjuvanted with AddaVax, and were boosted 28 days later (Fig. 4a). Hamsters immunized with the VLP-S2 and VLP-S2_mutS2’_ generated high IgG antibody titers against the S ectodomain (Fig. 4b; Table 1). The vaccinated hamsters were intranasally inoculated with 10^3^ plaque-forming units (pfu) of NCGM02^45^ 51 days after the initial immunization. The hamsters were then sacrificed 3 days after infection and viral titers in their lungs and nasal turbinates were quantified by plaque assay. The mean viral titer in the lungs of hamsters immunized with VLP-S2 was nearly 100-fold lower than that of hamsters immunized with VLP-control (Fig. 4c). The mean viral titer in the lungs of hamsters immunized with VLP-S2_mutS2’_ was more than 7000-fold lower than that of control immunized hamsters (Fig. 4c). Characterization of viral titers in the nasal turbinates of the immunized hamsters also indicated that the multivalent S2 constructs provided partial protection against virus replication in the respiratory tissues (Fig. 4d). The mean viral titers in the nasal turbinates of hamsters immunized with VLP-S2 and VLP-S2_mutS2’_ were respectively 3- and 36-fold lower than that of hamsters immunized with VLP-control. It is possible that the increased efficacy seen with VLP-S2_mutS2’_ is due to the proteolytic cleavage of the S2 construct at the S2’ site *in vivo*, after immunization.

### Multivalent S2-based vaccines elicit cross-reactive antibodies

Next, the breadth of the immune response generated by VLP-S2 and VLP-S2_mutS2’_ was evaluated using ELISA. Immunization with the multivalent S2 constructs elicited high IgG antibody titers against the spike protein of not only the original Wuhan-Hu-1 SARS-CoV-2 (614D),^51^ but also against the spike proteins of the SARS-CoV-2 variant B.1.351, SARS-CoV-1, and the four endemic human coronaviruses HKU-1, OC43, NL63, and 229E (Fig. 4e and Table 1). This substantial cross-reactivity suggests that immunization with multivalent S2-based immunogens may be a promising strategy for eliciting a broadly protective response against coronaviruses.

### Characterization and optimization of the efficacy of VLP-S2_mutS2’_

Given the better efficacy provided by VLP-S2_mutS2’_ than VLP-S2 (Fig. 4c and d), we decided to use the VLP-S2_mutS2’_ construct for further characterization against a previous variant of concern, B.1.617.2, delta variant (hCoV-19/USA/WI-UW-5250/2021).^52^ We first assessed the efficacy of VLP-S2_mutS2’_ against B.1.617.2 using a similar prime/boost immunization regimen with AddaVax as the adjuvant as we first used against the early NCGM02 isolate (Fig. 4a). While there was a 35-fold and 2-fold decrease in the mean viral titers in nasal turbinate and lung tissues, respectively, for hamsters immunized with VLP-S2_mutS2’_ relative to controls, this difference was not statistically significant (Fig. 5a). We therefore optimized the immunization regimen to enhance the efficacy of the vaccine.

We first tested the effect of providing an extra vaccine dose, i.e., a third immunization with VLP-S2_mutS2’_, while retaining AddaVax as the adjuvant. Encouragingly, following a challenge with B.1.617.2, we now observed a statistically significant ∼90-fold decrease in the mean lung viral titers of hamsters immunized with VLP-S2_mutS2’_ compared to those of control vaccinated hamsters and a ∼11-fold decrease in the mean nasal turbinate viral titers relative to controls (Fig. 5b).

Having demonstrated the greater efficacy provided by an additional dose of the vaccine, we next tested the effect of different adjuvants, which can greatly influence the magnitude and quality of the immune response.^53^, ^54^, ^55^, ^56^ For these experiments, we assessed the efficacy of VLP-S2_mutS2’_ against B.1.617.2 using a prime/boost immunization regimen (Fig. 4a), but using the adjuvants – QS-21, AddaS03 (a commercially available adjuvant system similar to GSK's AS03) plus poly I:C (AS03 + pIC), and R848 (Fig. 5c and d). We observed a statistically significant decrease in lung viral titers for hamsters immunized with VLP-S2_mutS2’_ relative to controls, when using QS-21 or AS03 + pIC as adjuvants (33-fold and 127-fold, respectively; Fig. 5c). In contrast, no significant difference in lung viral titers was seen when using R848 as an adjuvant. We also observed a significant decrease in nasal turbinate viral titers for hamsters immunized with VLP-S2_mutS2’_ relative to controls when using QS-21 or AS03 + pIC as adjuvants (∼18-fold and ∼195-fold, respectively; Fig. 5d). Based on these results (Fig. 5b–d), we selected a three-dose immunization regimen, with a mixture of AS03 + pIC as adjuvants, for further characterization of the breadth of protection.

### VLP-S2_mutS2’_ demonstrates efficacy against challenges with SARS-CoV-2 variants of concern and pangolin coronaviruses

We first characterized the breadth of the antibody response elicited by the optimal immunization regimen (3 doses; AS03 + pIC) by ELISA. Consistent with the high degree of conservation in the S2 domain, immunization with VLP-S2_mutS2’_ elicited high IgG antibody titers against early SARS-CoV-2 spike proteins (either with aspartate (D) or glycine (G) at position 614 [S-614D or S-614G, respectively), spike proteins of variants (B.1.617.2, B.1.351, BA.1 and BA.2), as well as against the spike proteins of other sarbecoviruses including a bat coronavirus (SARS-like coronavirus, RsSHC014), a pangolin coronavirus (Pg CoV) (BetaCoV/pangolin/Guandong/1/2019), and SARS-CoV-1 (Fig. 6a). Moreover, we also observed high IgG antibody titers against the spike protein of the endemic human coronavirus NL63 (Fig. 6a).

Next, we tested the efficacy of this immunization regimen against a challenge with the SARS-CoV-2 variants, hCoV-19/USA/MD-HP01542/2021 (B.1.351, beta), hCoV-19/USA/WI-WSLH-221686/2021 (B.1.1.529 BA.1, Omicron), and B.1.617.2 (delta). For each variant challenge virus, we observed a statistically significant decrease in lung viral titers (Fig. 6b) for hamsters immunized with VLP-S2_mutS2’_ compared to control vaccinated hamsters. There was a greater than 6000-fold decrease in lung viral titers for hamsters immunized with VLP-S2_mutS2’_ relative to controls following a challenge with BA.1 (which shows extensive mutations in the S1 domain), highlighting the effectiveness of targeting the immune response towards the conserved S2 domain. We also observed significant decreases in mean nasal turbinate viral titers (Fig. 6c) on day 3 after infection with these variants for hamsters immunized with VLP-S2_mutS2’_ compared to control immunized hamsters.

The optimal immunization regimen was also very effective at reducing viral titers in respiratory tissues following a challenge with Pg CoV, BetaCoV/pangolin/Guandong/1/2019. We were unable to detect replicating Pg CoV in the lungs of vaccinated hamsters (limit of detection 1.3 log_10_ pfu/g) while Pg CoV replicated to ∼10^5^ to 10^7^ pfu/g in the lungs of control unvaccinated hamsters (Fig. 6d). In the nasal turbinates of hamsters, Pg CoV replicated better compared to the lungs, and vaccination with VLP-S2_mutS2’_ reduced virus titers in the nasal turbinates by 100-fold (Fig. 6d).

Sera from hamsters immunized with VLP-S2_mutS2’_ using the optimal immunization regimen showed neutralization activity *in vitro* against SARS-CoV-2/UT-HP095-1N/Human/2020/Tokyo, an early S-614D isolate in a focus reduction neutralization test (FRNT) assay. Sera from hamsters immunized with VLP-S2_mutS2’_ also showed neutralization activity *in vitro* against the SARS-CoV-2 B.1.351, B.1.617.2, and BA.1 variants, as well as Pg-CoV in the FRNT assay (Fig. 6f).

### Immunization with VLP-S2_mutS2’_ demonstrates efficacy in mice against a SARS-CoV-2 challenge in mice and elicits a broadly neutralizing antibody response

Recently, Ng et al.^57^ reported a DNA-vaccine-based approach to elicit S2-targeted immunity in mice. Sera from S2-immunized mice demonstrated broad neutralizing activity *in vitro*. While this study did not demonstrate protection from a challenge with more recent variants of concern such as delta and BA.1, or with non-SARS-CoV-2 sarbecoviruses, SARS-CoV-2-S2–vaccinated K18-hACE2 mice challenged with SARS-CoV-2 Wuhan and Alpha isolates showed a 0.9 and 1.1 log reduction in SARS-CoV-2 E copies in the lungs on day 4, respectively, compared with unvaccinated controls. In our mouse study, while a mouse-adapted SARS-CoV-2 (an early Wuhan-like isolate)^46^ replicated to > 10^7^ pfu/g in the lungs of control unvaccinated BALB/c mice (Fig. 7a), we were unable to detect replication of the mouse-adapted virus in the lungs of vaccinated mice (limit of detection 1.3 log_10_ pfu/g). Sera from mice obtained after a single immunization with VLP-S2_mutS2’_ also showed neutralization activity *in vitro* against three SARS-CoV-2 variants and Pg-CoV in an FRNT assay (Fig. 7b).

## Discussion

Despite the high efficacy of licensed vaccines against the original SARS-CoV-2 strain, studies have shown reduced protection against recent variants, mostly due to the high number of mutations found in areas of the S protein targeted by these vaccines. As a result, there has been a growing interest in improving the breadth of protection provided by vaccines. While near-term interest is focused on vaccines that provide protection against SARS-CoV-2 variants (pan-SARS-CoV-2), there is also an interest in developing vaccines that protect against all sarbecoviruses (pan-sarbecovirus) and ultimately vaccines that provide protection against all betacoronaviruses (pan-betacoronavirus). There are two main strategies for designing vaccines that elicit a broadly protective antibody response. One strategy, which has been used successfully with seasonal influenza vaccines, is based on using a mixture of different antigens. For instance, groups are pursuing the design of nanoparticle-based coronavirus vaccines that use a mixture of different receptor-binding domain (RBD) antigens.^58^, ^59^, ^60^ Cohen et al. have reported the design of mosaic nanoparticle vaccines – vaccines displaying multiple RBDs on the same nanoparticle.^58^^,^^59^ Mosaic nanoparticles based on a mixture of 8 different clade 1 and clade 2 sarbecovirus RBD antigens protected against challenges with both SARS-CoV-1 and SARS-CoV-2 and also showed broad neutralization activity *in vitro*. Similarly, Walls et al. showed that mosaic nanoparticles based on a mixture of 4 different sarbecovirus RBDs could protect against a challenge with SARS-CoV-1, although the SARS-CoV-1 RBD was not a component of the vaccine.^60^ While these results are promising and consistent with an approach that has worked with seasonal influenza vaccines, concerns include the large number of antigens required to provide broad protection as well as the plasticity of RBD domain and the possible emergence of new vaccine-evading RBD variants.

A second approach is to focus on parts of the S protein that are more conserved, which might enable the design of broadly protective vaccines without using mixtures of 4 or 8 antigens. To that end, we have developed, characterized, and optimized immunogens based on the conserved S2 domain of SARS-CoV-2. The optimal immunization regimen – three doses of the VLP-S2_mutS2’_ construct with AS03 + pIC as an adjuvant demonstrated efficacy in hamsters against challenges with SARS-CoV-2 variants as well as a pangolin coronavirus.

While sera from immunized animals in our study neutralized the viruses *in vitro*, other mechanisms, such as Fc effector functions, may also contribute to the efficacy provided by immunization with the multivalent S2 constructs. Fc effector functions have previously been identified as a mechanism by which S2-specific antibodies provide protection.^29^ In addition, antibodies targeting the S2-analogous region of the influenza protein hemagglutinin (the stalk domain) are known to provide protection through Fc effector functions.^61^ We will investigate the contribution of these non-neutralizing mechanisms to protection in future work.

We also note that this study primarily used hamsters to evaluate the immunogenicity and efficacy of our S2-based vaccine construct because hamsters are naturally susceptible to infection by SARS-CoV-2 without the requirement of virus adaptation or the need of human ACE2 expressing transgenic hamsters. We show similar results for the S2-based vaccine construct in the mouse model using a mouse-adapted SARS-CoV-2 isolate.^46^ However, these results in hamsters and mice may not translate to humans due to differences in viral replication and immune response between species. Translation of this work will require human clinical trials. It is therefore important to consider potential obstacles to clinical translation. One potential concern is the expression yield of the S2_mutS2’_ construct. Expression of this construct in insect cells could be considered if necessary to improve the expression yield. Further optimization would also be needed to develop a manufacturing process that is compliant with cGMP (current Good Manufacturing Practices). However, vaccines based on other nanoscaffolds are in clinical trials, supporting the feasibility of our nanoparticle-based vaccines.^62^^,^^63^

In summation, we have developed a vaccine based on the conserved S2 domain of SARS-CoV-2 (Table S2) and have characterized and optimized the immunogenicity and efficacy of this vaccine to significantly reduce virus replication in the respiratory tissues of vaccinated rodents. We demonstrated that multivalent S2 constructs are capable of eliciting a broadly cross-reactive immune response that protects against multiple sarbecoviruses including an early isolate of SARS-CoV-2, SARS-CoV-2 variants (beta, delta, and omicron), and a pangolin coronavirus. Based on these promising results, the S2 subunit should be considered in the development of next-generation coronavirus vaccines designed to protect against future SARS-CoV-2 variants and other zoonotic coronaviruses with pandemic potential.

## Contributors

All authors (P.J.H., S.J.F, K.L., M.K., T.M., T.A., J.E.Y., Y.J.H., R.B. E.R.W., Y.K., and R.S.K.) were involved in the study design. P.J.H., S.J.F., K.L., M.K., T.A., and J.E.Y. performed data collection and data analysis. P.J.H., S.J.F., K.L., and R.S.K. wrote the manuscript. P.J.H and K.L. have access to and verified the data. All authors read and approved the final manuscript.

## Data sharing statement

The data needed to support the conclusions of this study have been included in the paper. The data that underly the graphs and charts within the paper are included in Supplementary Data. All other data supporting the conclusions of this study are available from the corresponding authors upon reasonable request.

## Declaration of interests

YK has received unrelated funding support from 10.13039/501100002336Daiichi Sankyo Pharmaceutical, 10.13039/501100019949Toyama Chemical, Tauns Laboratories, Inc., Shionogi & Co. Ltd., Otsuka Pharmaceutical, KM Biologics, Kyoritsu Seiyaku, Shinya Corporation, and 10.13039/100014400Fujirebio. PJH, SJF, KL, YK, and RSK are inventors on a patent application related to this work. The other authors declare no competing interests.
