# Supplementary material for: Multivalent S2-based vaccines provide broad protection against SARS-CoV-2 variants of concern and pangolin coronaviruses
Source: eBioMedicine. 2022 Nov 11;86:104341. doi: 10.1016/j.ebiom.2022.104341 (PMC9651965; doi:10.1016/j.ebiom.2022.104341)
Supplement: Supplementary File S1 [file mmc2.pdf]

Supplementary Information

Multivalent S2-Based Vaccines Provide Broad Protection Against SARS-CoV-2 Variants of Concern and Pangolin Coronaviruses

Peter J. Halfmann[1,†], Steven J. Frey[2,†], Kathryn Loeffler[2,†], Makoto Kuroda[1], Tadashi Maemura[1],Tammy Armbrust[1], Jie E. Yang[3,4,5], Yixuan J Hou[6], Ralph Baric[6,7], Elizabeth R. Wright[3,4,5], Yoshihiro Kawaoka[1,8]*, Ravi S. Kane[2,9]*

[1]Influenza Research Institute, Department of Pathobiological Sciences, School of Veterinary Medicine, University of Wisconsin, Madison, WI, 53711, USA

[2]School of Chemical & Biomolecular Engineering, Georgia Institute of Technology, Atlanta, Georgia, 30332, USA

[3]Department of Biochemistry, University of Wisconsin, Madison, WI, 53706, USA

[4]Cryo-EM Research Center, Department of Biochemistry, University of Wisconsin, Madison, WI, 53706, USA

[5]Midwest Center for Cryo-Electron Tomography, Department of Biochemistry, University of Wisconsin, Madison, WI, 53706, USA

[6]Department of Epidemiology, University of North Carolina at Chapel Hill, Chapel Hill, NC, 27599, USA.

[7]Department of Microbiology and Immunology, University of North Carolina at Chapel Hill, Chapel Hill, NC, 27599, USA

[8]Division of Virology, Department of Microbiology and Immunology, Institute of Medical Science, University of Tokyo, Tokyo 108-8639, Japan

[9]Wallace H. Coulter Department of Biomedical Engineering, Georgia Institute of Technology, Atlanta, Georgia, 30332, USA

† These authors contributed equally

* To whom correspondence should be addressed:
yoshihiro.kawaoka@wisc.edu, ravi.kane@chbe.gatech.edu

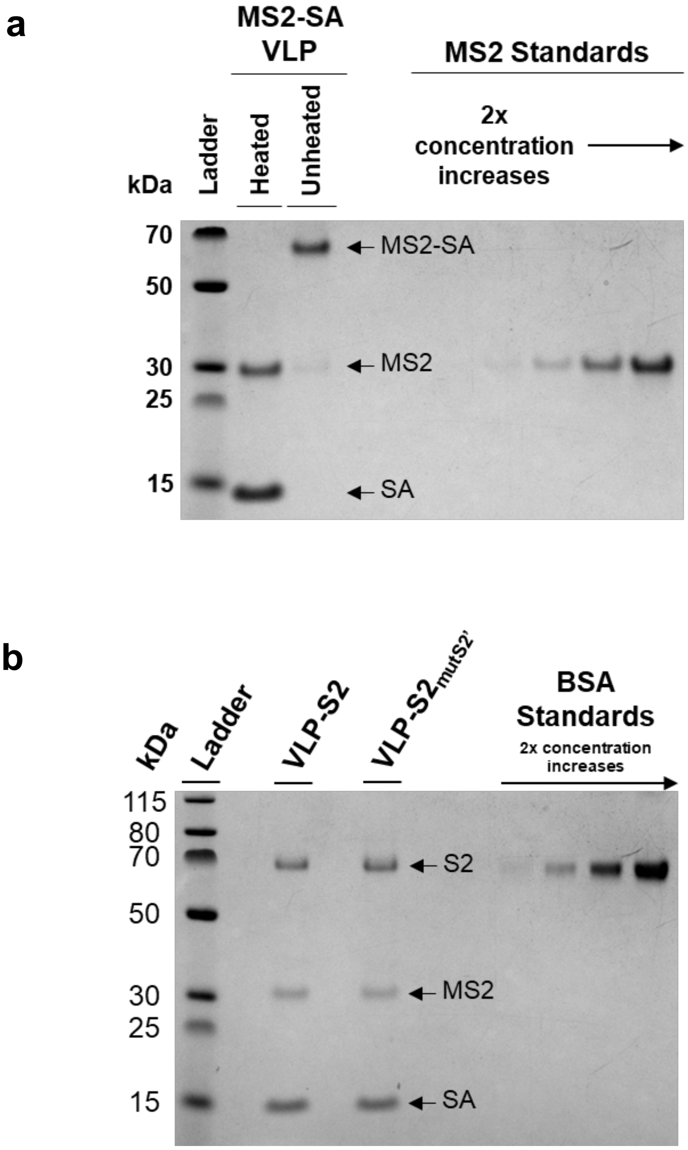

Fig. S1. Characterization of MS2 to SA to S stoichiometry using SDS-PAGE. (**a**) Amount of MS2 in heated MS2-SA VLP was compared to amount of MS2 in unheated MS2-SA VLP to determine that approximately 78 percent of MS2 was bound to SA. Excess biotin was added to the unheated sample to occupy all unoccupied biotin binding sites prior to the addition of SDS. β-mercaptoethanol was added to all samples. (**b**) The intensity of bands corresponding to S2 and MS2 in VLP-S2 and VLP-$S2_{mutS2'}$ were compared to BSA standards and quantified to determine that approximately 30 S2 molecules were displayed on each MS2-SA VLP. S2 was deglycosylated with PNGase F and all samples were heated with β-mercaptoethanol and LDS sample buffer. The unprocessed gels are shown in Fig. S2.

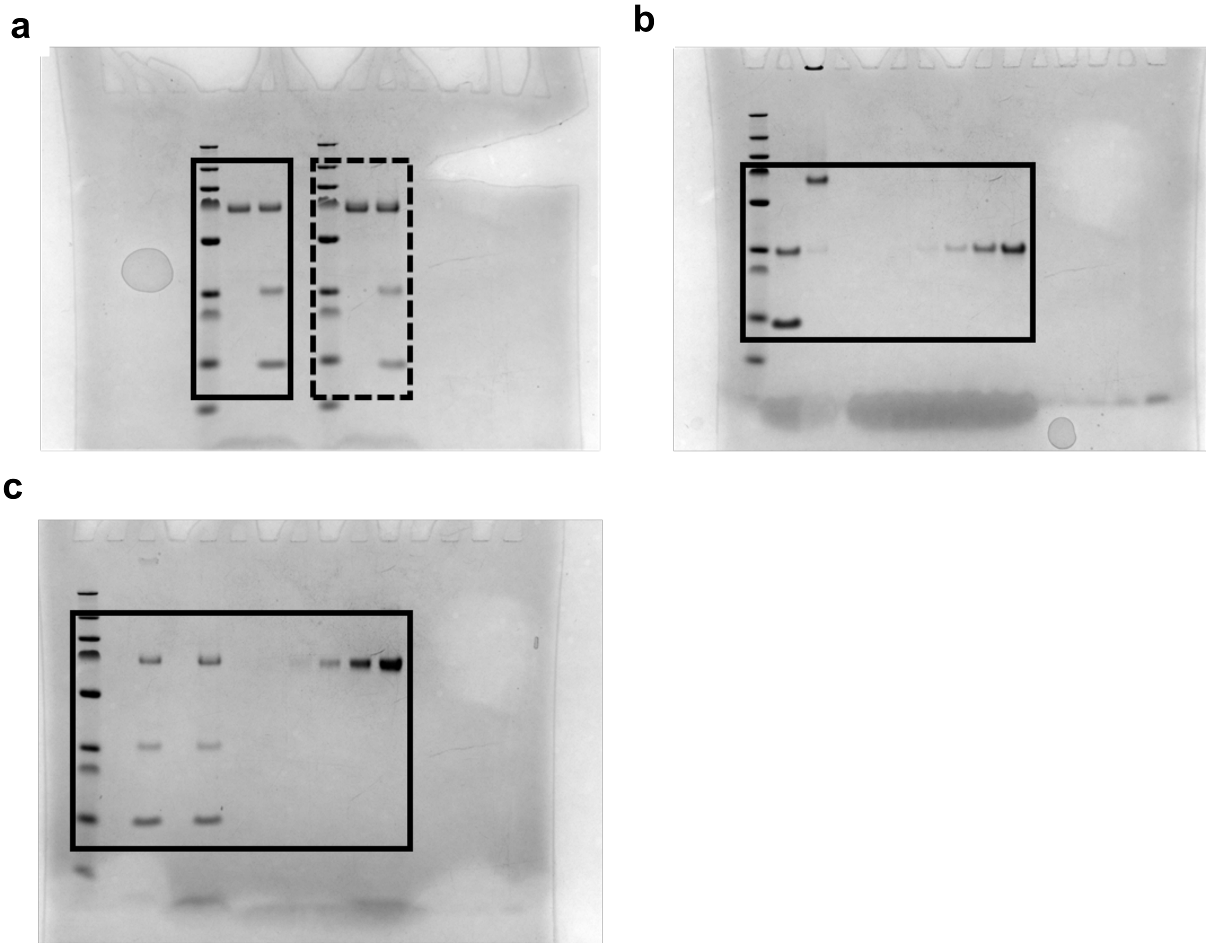

Fig. S2. Unprocessed SDS-PAGE gel images. Unprocessed SDS-PAGE gel images, cropped versions of which appear in (**a**) Fig. 2a (solid rectangle), Fig. 3a (dashed rectangle), (**b**) Fig. S1a, and (**c**) Fig. S1b.

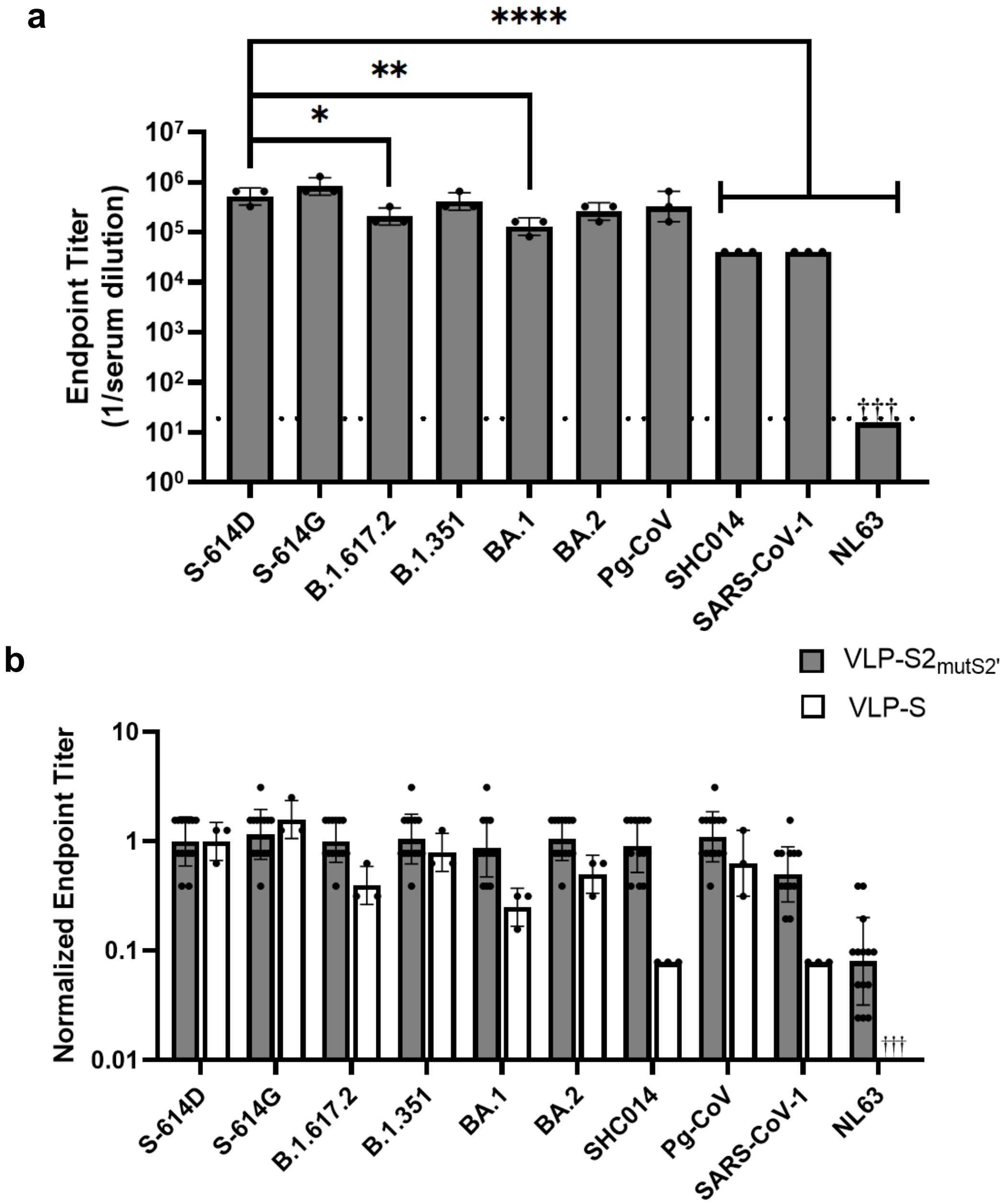

Fig. S3. Characterization of antibody response to VLP-S and VLP-S2$_{mutS2'}$. (**a**) IgG antibody endpoint titers induced by immunization with VLP-S against S proteins from various coronaviruses (geometric mean with geometric SD, n = 3, biological replicates = 3: sera from 3 hamsters) **P* = 0.0046, ***P* = 0.0017, *****P* < 0.0001 [Ordinary One-Way ANOVA]. † - Endpoint titers below the detection limit (<20). Detection limit (dotted line) = 1:20 dilution. (**b**) Antibody titers induced by immunization with VLP-S2$_{mutS2'}$ or VLP-S normalized to the S-614D response. (geometric mean with geometric SD, biological replicates and n = 14 for VLP-S2$_{mutS2'}$: sera from 14 hamsters, biological replicates and n = 3 for VLP-S: sera from 3 hamsters). † = Below the limit of detection.

Table S1. Protein sequences

**MS2-AviTag**	MASNFTQFVLVDNGGTGDVTVAPSNFANGVAEWISSNSRSQAYKVTCSVRQSS<br>AQNRKYTIKVEVPKVATQTVGGVELPVAAWRSYLNMELTIPIFATNSDCELIV<br>KAMQGLLKDGNPIPSAIAANSGIYASNFTQFVLVDNGGGLNDIFEAQKIEWHE<br>TGDVTVAPSNFANGVAEWISSNSRSQAYKVTCSVRQSSAQNRKYTIKVEVPKV<br>ATQTVGGVELPVAAWRSYLNMELTIPIFATNSDCELIVKAMQGLLKDGNPIPS<br>AIAANSGIY
**S2**	METDTLLLWVLLLWVPGSTGDSVASQSIIAYTMSLGAENSVAYSNNSIAIPTN<br>FTISVTTEILPVSMTKTSVDCTMYICGDSTECSNLLLQYGSFCTQLNRALTGI<br>AVEQDKNTQEVFAQVKQIYKTPPIKDFGGFNFSQILPDPSKPSKRSPIEDLLF<br>NKVTLADAGFIKQYGDCLGDIAARDLICAQKFNGLTVLPPLLTDEMIAQYTSA<br>LLAGTITSGWTFGAGPALQIPFPMQMAYRFNGIGVTQNVLYENQKLIANQFNS<br>AIGKIQDSLSSTPSALGKLQDVVNQNAQALNTLVKQLSSNFGAISSVLNDILS<br>RLDPPEAEVQIDRLITGRLQSLQTYVTQQLIRAAEIRASANLAATKMSECVLG<br>QSKRVDFCGKGYHLMSFPQSAPHGVVFLHVTYVPAQEKNFTTAPAICHDGKAH<br>FPREGVFVSNGTHWFVTQRNFYEPQIITTDNTFVSGNCDVVIGIVNNTVYDPL<br>QPELDSFKEELDKYFKNHTSPDVDLGDISGINASVVNIQKEIDRLNEVAKNLN<br>ESLIDLQELGKYEQGSGYIPEAPRDGQAYVRKDGEWVLLSTFLGGLNDIFEAQ<br>KIEWHEHHHHHH
**$S2_{mutS2'}$**	METDTLLLWVLLLWVPGSTGDSVASQSIIAYTMSLGAENSVAYSNNSIAIPTN<br>FTISVTTEILPVSMTKTSVDCTMYICGDSTECSNLLLQYGSFCTQLNRALTGI<br>AVEQDKNTQEVFAQVKQIYKTPPIKDFGGFNFSQILPDPSKPSGGSPIEDLLF<br>NKVTLADAGFIKQYGDCLGDIAARDLICAQKFNGLTVLPPLLTDEMIAQYTSA<br>LLAGTITSGWTFGAGPALQIPFPMQMAYRFNGIGVTQNVLYENQKLIANQFNS<br>AIGKIQDSLSSTPSALGKLQDVVNQNAQALNTLVKQLSSNFGAISSVLNDILS<br>RLDPPEAEVQIDRLITGRLQSLQTYVTQQLIRAAEIRASANLAATKMSECVLG<br>QSKRVDFCGKGYHLMSFPQSAPHGVVFLHVTYVPAQEKNFTTAPAICHDGKAH<br>FPREGVFVSNGTHWFVTQRNFYEPQIITTDNTFVSGNCDVVIGIVNNTVYDPL<br>QPELDSFKEELDKYFKNHTSPDVDLGDISGINASVVNIQKEIDRLNEVAKNLN<br>ESLIDLQELGKYEQGSGYIPEAPRDGQAYVRKDGEWVLLSTFLGGLNDIFEAQ<br>KIEWHEHHHHHH

Table S2. S2 Sequence Homology

	SARS-CoV-2 $S2_{mutS2'}$	B.1.351	B.1.617.2	BA.1	Pg-CoV
SARS-CoV-2 $S2_{mutS2'}$	100.0%	98.1%	98.3%	97.3%	96.9%
B.1.351	98.1%	100.0%	99.4%	98.5%	98.1%
B.1.617.2	98.3%	99.4%	100.0%	98.7%	98.3%
BA.1	97.3%	98.5%	98.7%	100.0%	97.3%
Pg-CoV	96.9%	98.1%	98.3%	97.3%	100.0%
